# Targeting Multidrug Resistance in Cancer: Impact of Retinoids, Rexinoids, and Carotenoids on ABC Transporters

**DOI:** 10.3390/ijms262211157

**Published:** 2025-11-18

**Authors:** Martina Čižmáriková, Viktória Háziková, Radka Michalková, Ondrej Franko, Beáta Lešková, Atila David Homolya, Juliana Gabzdilová, Peter Takáč

**Affiliations:** 1 Department of Pharmacology, Faculty of Medicine, Pavol Jozef Šafárik University, 040 11 Košice, Slovakia; viktoria.hazikova@student.upjs.sk (V.H.); ondrej.franko@student.upjs.sk (O.F.); beata.leskova@student.upjs.sk (B.L.); 2 Department of Radiotherapy and Oncology, East Slovakia Institute of Oncology, 041 91 Košice, Slovakia; atila.david.homolya@student.upjs.sk; 3 Department of Hematology and Oncohematology, Louis Pasteur University Hospital, Faculty of Medicine, Pavol Jozef Šafárik University, 040 11 Košice, Slovakia; juliana.gabzdilova@upjs.sk; 4 Department of Pharmacology and Toxicology, University of Veterinary Medicine and Pharmacy, Komenského 73, 041 81 Košice, Slovakia; peter.takac@uvlf.sk

**Keywords:** efflux, transporter, ABCB1, ABCC1, ABCC2, ABCG2, resistance, retinoid, rexinoid, carotenoid

## Abstract

The active efflux of drugs by adenosine triphosphate (ATP)-binding cassette (ABC) trans-porters, such as multidrug resistance protein 1 (MDR1/ABCB1), multidrug resistance-associated protein 1 and 2 (MRP1/ABCC1; MRP2/ABCC2), and breast cancer resistance protein (BCRP/ABCG2), is a well-established mechanism contributing to multidrug resistance (MDR). Interestingly, various vitamin A-based molecules have been found to influence the expression or function of these transporters. This work investigated the current evidence on the effects of retinoids, rexinoids, and carotenoids on ABC transporters and their potential to reverse MDR. Several studies indicated that these compounds could inhibit ABC transporter activity at non-toxic concentrations, either by downregulating gene/protein expression or by directly blocking efflux function. These effects were often associated with increased chemosensitivity to several conventional anticancer agents. Overall, the degree of inhibition varied depending on several factors, including compound type and their chemical modification, dose, incubation time, treatment timing, the type of target cells, method of transporter overexpression, and coadministration with other compounds. Although particular attention was paid to elucidating the underlying mechanisms, current knowledge in this area remains limited. Moreover, extensive in vivo and clinical studies validating these findings are still lacking, emphasizing the need for further research to evaluate their translational potential.

## 1. Introduction

In cancer treatment, chemoresistance describes the capacity of cancer cells to adapt and evade the effects of chemotherapy, often contributing to less favorable clinical outcomes. A major concern is multidrug resistance (MDR), where resistance to one anticancer drug extends to others with unrelated mechanisms of action [1].

One of the primary mechanisms underlying MDR in cancer is drug efflux mediated by adenosine triphosphate ATP-binding cassette (ABC) transporters, which reduces intracellular drug accumulation [2,3]. Among the seven ABC transporter subfamilies, ABCB, ABCC, and ABCG are most closely linked to MDR-related drug efflux [4]. Research has primarily focused on transporters such as ABCB1 (P-glycoprotein or multidrug resistance protein 1, MDR1), ABCC1 (multidrug resistance-associated protein 1, MRP1), and ABCG2 (breast cancer resistance protein, BCRP) [5,6,7,8,9], though other less-studied members like ABCC2 (multidrug resistance-associated protein 2, MRP2) are also under investigation [10,11,12]. These transporters can export a wide variety of substrates, including traditional chemotherapeutic drugs, such as anthracyclines, vinca alkaloids, and taxanes, as well as newer targeted agents like nilotinib, sorafenib, and vemurafenib [13]. Notably, ABC transporters are frequently overexpressed in tumor cells across various malignancies [13], including cancer stem cells (CSCs) [14]. Their classification, structural characteristics, localization, and functional roles have been extensively documented in the literature [5,7,9,13,15,16,17].

ABC transporters are subject to regulation at transcriptional, post-transcriptional, and post-translational levels. A wide range of factors can influence this regulation, including hormonal signals, inflammatory processes, oxidative stress, extracellular vesicles, circadian rhythms, microRNAs, and signaling pathways such as phosphoinositide 3-kinase/protein kinase B/mechanistic target of rapamycin (PI3K/Akt/mTOR) and wingless-related integration site/beta-catenin (WNT/β-catenin). Additionally, transcriptional regulators like nuclear factor kappa B (NF-κB), the pregnane X receptor (PXR), and the constitutive androstane receptor (CAR) are also involved in modulating their expression and function [18,19,20,21]. Beyond expression, protein function may be altered by variations in ATPase activity and changes in the physical properties of the plasma membrane, as well as by interactions, whether competitive, non-competitive, or allosteric, with various endogenous or exogenous substances [20].

At present, no clinically validated strategies exist to effectively overcome chemoresistance driven by ABC transporters, revealing a critical therapeutic challenge. Multiple experimental approaches are being explored to address this issue [2,16,21]. They include innovative drug delivery platforms such as nanoparticles, antibody-drug conjugates, targeted treatments, ultrasound waves and photothermal techniques, as well as genetic interventions targeting the genes responsible for ABC transporter expression. In parallel, there is a strong focus on discovering effective, selective, and safe inhibitors of these transporters. Although numerous inhibitors and modulators of ABC transporters (e.g., verapamil, valspodar, and tariquidar) have been developed in the past, none have been successfully implemented in clinical practice owing to their low selectivity, high toxicity, unpredictable pharmacokinetic interactions, and limited clinical efficacy [17,22]. Recently, some newly developed targeted small molecules, such as furmonertinib, a third-generation epidermal growth factor receptor tyrosine kinase inhibitor, has shown, in addition to its antitumor effects, the ability to inhibit ABCB1 and ABCG2 [23]. Natural compounds are of particular interest due to their potential for enhanced specificity, reduced toxicity, and ability to interact with multiple molecular targets [4,24,25,26,27]. Despite their diverse chemical structures, many phytochemicals and their synthetic derivatives have been shown to inhibit ABC transporters. These include polyphenols such as resveratrol [28], quercetin [29], curcumin, salvianolic acid B, and epigallocatechin gallate [4]; chalcones [30,31,32]; terpenoids such as ursolic acid, lupeol, tanshinone IIA, cryptotanshinone, dihydrotanshinone, and α-turmerone [4]; as well as various alkaloids [33]. Other well-known natural ABC transporter inhibitors and their derivatives include antimalarial agents [34], marine-derived compounds [35,36], and traditional Chinese herbal medicines, such as tetrandrine from *Tinospora crispa* [37], and terpenoids and polysaccharides from *Ganoderma lucidum* [31]. Moreover, several natural compounds have demonstrated the ability to interact with ABC transporters present in drug-resistant bacteria, such as methicillin-resistant *Staphylococcus aureus* [38].

Recent studies have shown that certain vitamins, particularly lipophilic ones, can also modulate efflux transporter function and help overcome drug resistance [39,40,41,42]. This effect has also been observed in vitamin A-derived compounds, including retinoids, rexinoids, and carotenoids. Notably, some authors regard rexinoids as distinct from retinoids due to their specific receptor interaction mechanisms [43,44]. Further details of these substances are described in the following chapters.

**Figure 1:**
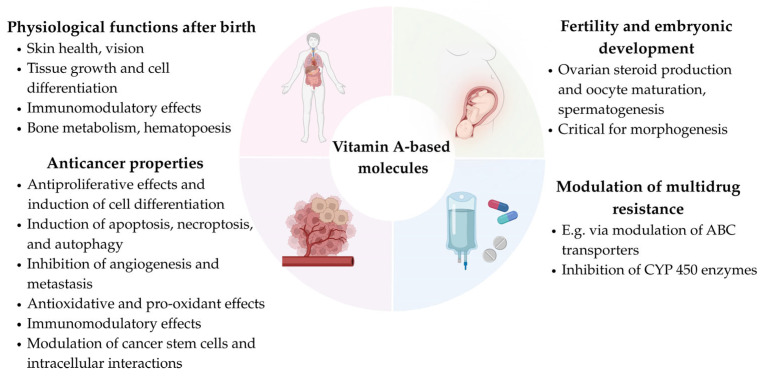
Biological functions of vitamin A-based molecules.

Collectively, retinoids, rexinoids, and carotenoids are either natural or synthetic agents involved in various biological processes [45,46,47,48,49,50,51,52,53,54,55,56] (Figure 1). Some of these compounds are used or recommended for the treatment and prevention of various medical conditions, particularly skin, eye, and cancer-related diseases [51,56,57] and are also found in nutritional supplements and cosmetics [54,58,59,60]. Additionally, selected carotenoids are used as natural colorants and food additives, favored over synthetic alternatives for their advantageous properties [61,62].

**Figure 2:**
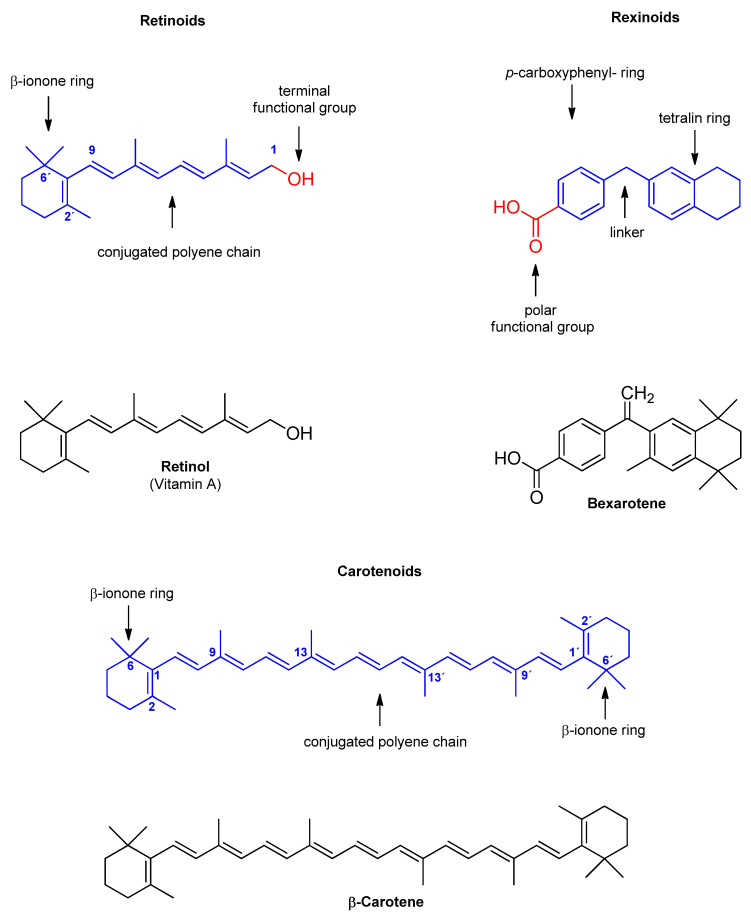
Overview of the core structural backbone of retinoids, rexinoids and carotenoids and their representative compounds. The characteristic backbone of each class is highlighted in blue, with labels indicating key structural features, while functional groups are marked in red. Representative compounds (retinol, bexarotene, β-carotene) of retinoids, rexinoids and carotenoids are also shown to exemplify each class.

Figure 2 illustrates the core structural scaffolds of the three classes of vitamin A discussed. Representative compounds (retinol, bexarotene, β-carotene) of retinoids, rexinoids and carotenoids are also shown to exemplify each class.

The aim of this study was to evaluate the current evidence on the effects of retinoids, rexinoids and carotenoids on ABC transporters, particularly regarding their ability to modulate or overcome drug resistance to conventional anticancer agents. To the best of our knowledge, no comprehensive study has yet addressed this topic in a systematic manner. The outcome of this work is intended to support and guide further investigations into the role of these promising compounds in modulating ABC transporter activity.

## 2. Methodology and Main Results

A comprehensive literature search was conducted across several major scientific databases, including PubMed, ScienceDirect, and Google Scholar, to identify relevant studies. The search covered publications from January 2001 to August 2025, using keywords related to the topic of interest (e.g., ABCB1, ABCC1, ABCG2, BCRP, carotenoid, MDR1, MRP1, P-glycoprotein, retinoid, rexinoid, vitamin A). Boolean operators (AND, OR) were used to refine and expand the search where appropriate.

Only peer-reviewed papers published in English were included in this study. Conference abstracts, editorials, and book chapters were excluded. As no clinical trials were identified during the search of commonly used clinical trial registries, including ClinicalTrials.gov, WHO International Clinical Trials Registry Platform, and, the Cochrane Central Register of Controlled Trials, the scope of this study is limited to in vitro findings. The extracted information included study identifiers, bioactive agents and their origins, cell types and model systems used, selected efflux transporters, experimental conditions (e.g., concentrations, incubation times), key outcomes (e.g., gene expression, protein expression, and efflux activity), and mechanistic insights, where available. In addition to these criteria, personal academic interest also influenced the inclusion of specific works. The results were synthesized qualitatively, with emphasis on recurring patterns.

During the period examined, the effects of various retinoids (n = 10), rexinoids (n = 1), and carotenoids (n = 41) on ABC transporters were investigated across 27 studies. These investigations encompassed assessment of changes in gene and protein expression, transporter functionality (mainly indirectly through monitoring the accumulation of ABC transporter substrates), protein conformation, and the potential to reverse chemoresistance. Notably, most of the research focused on the ABCB1 transporter, only less than half of studies (n = 11; 40.74%) addressing additional transporters such as ABCC1, ABCC2 or ABCG2 [63,64,65,66,67,68,69,70,71,72,73]. Approximately a third of the studies probed the mechanistic underpinnings of transporter modulation or chemoresistance reversal, with only a single study utilizing in silico methods to identify potential regulatory factors. Similarly, only two investigations have assessed effects of vitamin A-based molecules on ABC transporters within cells displaying a CSC phenotype [72,73]. To our knowledge, no clinical trials have yet addressed this issue. Nevertheless, certain studies have evaluated patient samples either following in vivo compound administration [74] or using ex vivo-treated samples [65,75]. A single study also assessed the safety profile of the compound under investigation [74]. Overall, studies have been conducted on cancer cells across a range of malignancies, including leukemias [74,75,76,77,78,79,80], lymphomas [81,82], colorectal [46,64,71,79,83,84,85], gastric [86] and hepatocellular carcinomas [46,70], as well as breast [63,70,82,87,88], ovarian [65,66,69,70], cervical [67], lung [67] and testicular [72] cancers, as well as on other cell models with transfection of genes for ABC transporters [67,68] or healthy cells [89]. Notably, studies performed on cells of human origin predominated. The number of publications has declined since 2015, with only ten studies published during this period.

**Figure 3:**
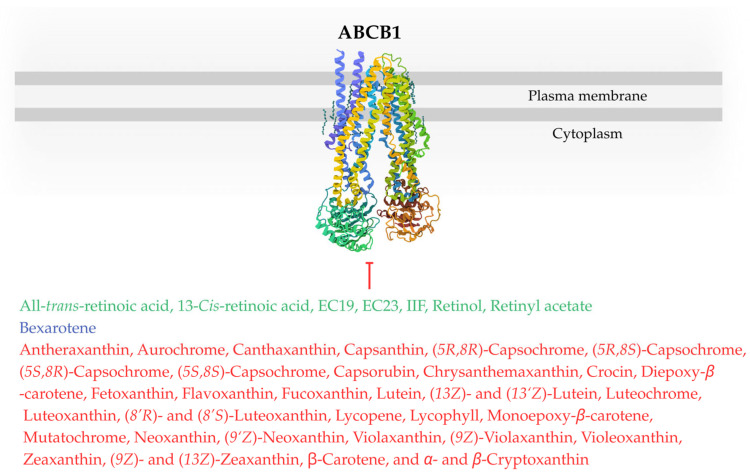
Potential inhibitors of ABCB1 transporter. The figure presents compounds demonstrating inhibitory activity against the respective transporter in at least one experimental context. Retinoids are depicted in green, rexinoid in blue, and carotenoids in red in the figure.

**Figure 4:**
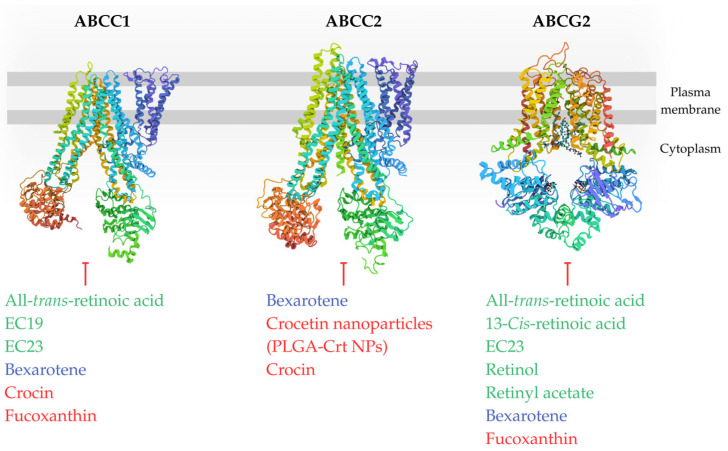
Potential inhibitors of ABCC1, ABCC2, and ABCG2 transporters. The figure presents compounds demonstrating inhibitory activity against the respective transporters in at least one experimental context. Retinoids are depicted in green, rexinoid in blue, and carotenoids in red in the figure.

**Figure 5:**
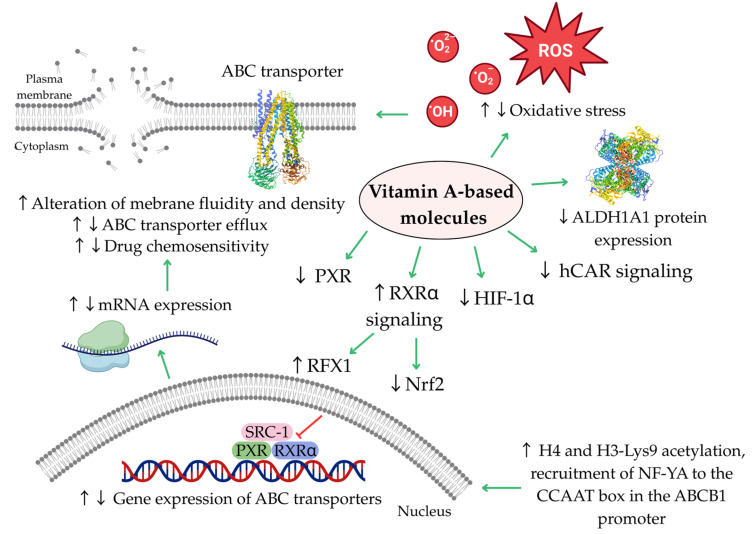
Biological functions of vitamin A-based molecules. Putative mechanisms potentially underlying the regulation of ABC transporters by vitamin A-based molecules. Further details regarding these mechanisms are provided in the relevant sections of the manuscript. Green arrows indicate activation; red arrow indicates inhibition; The symbols ↑ and ↓ indicate increased and decreased levels. ALDH1A1, aldehyde dehydrogenase 1 family member 1A; hCAR, human constitutive androstane receptor; HIF-1α, hypoxia-inducible factor 1α; Nrf2, nuclear factor erythroid 2-related factor 2; NF-YA, nuclear transcription factor Y alpha; PXR, pregnane X receptor; RFX1, regulatory factor X1; ROS, reactive oxygen species; RXR, retinoid X receptor; SRC-1, steroid receptor coactivator-1.

Next section details the impact of retinoids, rexinoids, and carotenoids on ABC transporters and the resulting modulation of chemoresistance observed across different cell types, with Figure 3 and Figure 4 showing potential inhibitors of individual transporters. Each paragraph of main text is ended by a concise summary of the established state of knowledge based on preceding research. Original Figure 1, Figure 3, Figure 4 and Figure 5 were created using BioRender (2025) (https://www.biorender.com/) and Canva software (2025) (https://www.canva.com/).

## 3. Retinoids and ABC Transporters

The term retinoid is commonly applied to a range of compounds that have structural or biological activities like vitamin A (retinol) [90]. Main endogenously occurring forms found in animals include retinol (all-*trans*-retinol), retinaldehyde (retinal, all-*trans*-retinal), all-*trans*-retinoic acid (ATRA, tretinoin), 9-*cis*-retinoic acid (alitretinoin), 13-*cis*-retinoic acid (isotretinoin), retinyl esters (primarily retinyl palmitate and retinyl acetate), 4-oxo-retinol, 4-oxo-retinoic acid, 14-hydroxy-4,14-retro-retinol, anhydroretinol, while the food (mainly of animal origin, such as liver, egg yolk, and milk fat in dairy products) contains mainly esters and a smaller amount of retinol [56,90,91]. Additionally, retinyl acetate and retinyl palmitate are frequently utilized as fortifying agents in food products, including butter, milk, cooking oils, and margarine.

Retinoids, whether naturally occurring or synthetically produced (e.g., retinol, retinal, tretinoin, alitretinoin, isotretinoin, etretinate, acitretin, adarotene, tazarotene, adapalene, and trifarotene), are characterized as fat-soluble, unsaturated hydrocarbons with a conserved β-ionone ring (a six-membered cyclohexene ring), an extended conjugated polyene chain, and a C15 terminal functional group that defines their chemical reactivity and biological activity [90] (Figure 2). Notably, their effects are mediated through retinoic acid receptors (RARs) and retinoid X receptors (RXRs), leading to transcriptional regulation of target genes [92].

Retinol and its derivatives play a central role in dermatology and cosmetic science, where they are used to treat skin disorders and improve skin quality (e.g., acne, psoriasis, photoaging, hyperpigmentation) [54,56,93], but specific compounds have already shown promising results in the treatment of certain malignancies. For example, ATRA is successfully used to treat acute promyelocytic leukemia (APL) [94], 9-*cis*-retinoic acid’s immune-modulatory properties are particularly important in treating conditions such as Kaposi’s sarcoma or other skin malignancies [95,96], and 13-*cis*-retinoic acid is used off-label to treat high risk neuroblastoma [97]. Retinoids are being actively studied for potential use in other conditions, such as gastric cancer, prostate cancer, T-cell acute lymphoblastic leukemia or multiple myeloma [98,99,100,101].

Overall, retinoids exhibit anticancer activity mainly by suppressing proliferation, migration, and invasion, while promoting apoptosis and cell differentiation [91]. Their mechanisms also involve, to a lesser extent, antioxidant effects [56]. Moreover, retinoids are known to influence mitochondrial dynamics, microRNA regulation, CSC behavior, and immune modulation [91].

Conversely, there is also evidence of contradictory outcomes. The clinical CARET study (Carotene and Retinol Efficacy Trial), conducted in the 1990s, showed an increased incidence of lung cancer in smokers and individuals exposed to asbestos [102].

While earlier study described an increase in ABCB1 expression at both the gene and protein levels in neuroblastoma cells following exposure to compound ATRA [103], several subsequent findings suggest that specific retinoids may also downregulate ABC transporter expression or activity, or improve cellular sensitivity to selected chemotherapeutic drugs [65,68,71,74,79,84,85].

The effects of individual retinoids on ABC transporters are listed in Table 1, and their chemical structures are shown in Figure S1.

### 3.1. Retinoids and ABC Transporters in Hematological Malignancies

The significance of retinoids in hematological malignancies is underscored by the introduction of ATRA as a pivotal therapeutic agent for APL in the late 20th century [104,105,106], and the combination of ATRA and arsenic trioxide (ATO) is currently recommended as standard therapy across all age groups [94]. Hyperacute, life-threatening leukemia has thus been transformed into a highly treatable hematologic malignancy, characterized by a high rate of complete remission [94,107]. In the treatment of APL, ATRA is primarily recognized for its ability to induce differentiation of leukemic cells. Recent studies suggest that this differentiation is largely mediated through the activation of autophagy pathways [108,109]. Interestingly, a recent study demonstrated that multidrug-resistant human APL (HL-60/RS) cells overexpressing ABCB1, ABCC1, and ABCG2 proteins also exhibit marked resistance to ATO, further emphasizing the importance of identifying approaches to overcome efflux-mediated drug resistance [110].

To date, retinoids have not been incorporated into the standard treatment protocols for other hematological malignancies beyond APL. However, several studies have also investigated the role of retinoids in relation to ABC efflux transporters across various types of hematological malignancies due to the confirmed elevated gene or protein expression of these transporters, especially ABCB1, in both leukemia and lymphoma cells [111,112].

Interestingly, among newly diagnosed acute myeloid leukemia (AML) patients treated with ATRA for three days before starting induction chemotherapy (n = 27), myeloblasts collected from bone marrow or peripheral blood demonstrated significantly higher in vitro rhodamine 123 (Rh-123) accumulation. In contrast, this effect was not seen in a comparable group of patients who underwent induction therapy without prior ATRA administration (n = 10) [74]. Additionally, pretreatment with ATRA significantly enhanced the complete remission rate compared to the control group; however disease-free survival and overall remission rates were comparable between the two groups. The therapy with ATRA was generally well tolerated, with a low incidence of adverse events. Only one patient experienced a common complication, ATRA syndrome, known also as differentiation syndrome [113], on the final day of drug administration. This presented as respiratory distress with hypoxemia, and chest X-ray confirmed bilateral diffuse pulmonary infiltrates. Notably, the patient’s leukocyte count remained below the threshold for leukocytosis. Discontinuation of ATRA and initiation of prednisone treatment resulted in clinical improvement.

Other researchers have reported that ATRA exerts differential effects on *ABCB1* gene expression and ABCB1 activity depending on the specific AML cell subtype [75]. The effect of this retinoid on potential transcription factors of *ABCB1* expression was also studied, namely the effect on mRNA expression of *early growth response 1 gene* (*Egr1*) and *Wilms’ tumor suppressor gene* (*WT1*). These transcription factors are frequently expressed in different cancers and are involved in tumor development [114,115]. While Egr1 upregulated *ABCB1* expression and increased chemoresistance [116,117], WT1 displayed its inverse regulation [118]. Another study reported simultaneous expression of *WT1* and *ABCB1* genes [119]. Furthermore, recent research indicated subtype-specific differences in *WT1* expression in AML [120], while upregulated *WT1* expression was associated with poor therapeutic response in another research [121]. The impact of ATRA on gene expression and transporter activity of ABCB1 was analyzed using real-time quantitative polymerase chain reaction (RT-PCR) and the Rh-123 efflux assay in two human AML cell lines, Kasumi-1 (harboring the t(8;21) translocation) and KG-1, as well as in selected populations of leukemic cells isolated from patient samples [75]. The tested patient samples were classified according to the older French-American-British (FAB) classification system, which categorizes AML subtypes based on the degree of leukemic cell differentiation and the predominant myeloid lineage they resemble, including M1 (AML without maturation, blastic AML type), M2 (AML with maturation) with t(8;21) karyotype, M3 (acute promyelocytic leukemia), and two samples M4 (acute myelomonocytic leukemia). In Kasumi-1 cells, which lack significant baseline expression of *ABCB1* and have a weak efflux activity, ATRA induced a measurable increase in *ABCB1* mRNA levels as early as one-hour post-incubation, accompanied by enhanced efflux activity after 72 h incubation. The effect of ATRA on ABCB1 is likely not mediated through *Egr1* and *WT1* in these cells, as *Egr1* mRNA expression was not detectable at any time point during the culture period and *WT1* expression remained stable throughout ATRA treatment. In contrast, in KG-1 cells, characterized by detectable basal *ABCB1* expression and strong efflux activity, ATRA treatment did not alter *ABCB1* mRNA levels at any of the evaluated time points (1, 3, 6, 24, 48, and 72 h), nor did it affect Rh-123 efflux. Stable *ABCB1* expression and efflux activity were maintained, even though *Egr1* mRNA expression decreased after ATRA treatment. On the other hand, *WT1* mRNA levels were stable upon incubation with the compound. ATRA also exhibited differential effects on *ABCB1* gene expression and Rh-123 efflux across patient-derived AML samples. In M1 blastic cells, characterized by high baseline Rh-123 efflux and non-quantifiable *ABCB1* mRNA expression levels (likely attributable to insufficient cell yield for reliable molecular analysis), ATRA had no observable impact on efflux activity. The M2 cells with t(8:21) karyotype, which showed moderate baseline *ABCB1* mRNA expression and intermediate efflux activity, demonstrated upregulation of both mRNA *ABCB1* expression and Rh-123 efflux following treatment. Similarly, M4 myelomonocytic cells, with low baseline *ABCB1* mRNA expression and minimal efflux activity, exhibited increased levels of both mRNA and efflux upon exposure to the compound. Conversely, in M3 promyelocyte-predominant cells, which had undetectable *ABCB1* mRNA expression (due to an insufficient cell yield for reliable molecular analysis) but moderate baseline efflux, treatment resulted in a reduction of Rh-123 efflux. Overall, the authors do not propose that ATRA exerts a stimulatory effect on ABCB1 activity. They interpret the elevated *ABCB1* expression in M2 cells as indicative of the immature nature of t(8;21) AML blasts, while in M4 cells, it is considered reflective of functionally active ABCB1 in differentiated monocytes. A partial contribution of ATRA to ABCB1 regulation through *Egr1* expression cannot be excluded in patient-derived samples. In M2 cells, an inverse relationship was observed between decreasing *Egr1* mRNA levels and increasing *ABCB1* mRNA expression during the 3–72 h incubation period, along with enhanced efflux activity following 72 h of treatment. In contrast, in M4 cells, an inverse correlation with ATRA exposure was evident only at specific time points and appeared to be sample-dependent. *WT1* mRNA expression, which was not detected (M2 cells) or was stable (M4 cells) after incubation with ATRA, probably does not affect *ABCB1* expression or function.

In line with earlier findings, subsequent study using semi-quantitative RT PCR demonstrated that ATRA can induce *ABCB1* mRNA expression in several hematological malignancy cell lines, including human acute T-cell leukemia (H9), chronic myeloid leukemia in blast crisis (K562), AML (KG-1), and APL (NB4), as well as in their RARα-transfected derivatives (H9/RAR, K562/RAR, KG-1/RAR, and NB4/RAR), irrespective of the baseline transporter expression levels [76]. Interestingly, no constitutive *ABCB1* mRNA expression was detected in H9 and NB4 cells, whereas the highest expression levels were observed in KG-1 cells. Apart from H9, Rh-123 efflux was augmented in these cell lines following 48 h of incubation with ATRA.

Furthermore, in acute promyelocytic leukemia NB4 cells lacking ABCB1 expression, ATRA increased *ABCB1* mRNA levels, protein expression, as well as transporter activity, particularly when combined with the histone deacetylase inhibitor depsipeptide (FK228) [77]. This effect was associated with enhanced acetylation of histones H4 and H3 at lysine 9 within the *ABCB1* promoter region, resulting from the recruitment of nuclear transcription factor Y alpha (NF-YA) to the CCAAT box. Elevated *ABCB1* mRNA levels were also observed in human AML cell lines naturally expressing ABCB1 (Kasumi-1 and Kasumi-6). The effect of combining retinoid with FK228 on doxorubicin efficacy in NB4 cells depended on the sequence of administration: doxorubicin cytotoxicity was markedly increased when ATRA/FK228 was administered after doxorubicin exposure, while a 24 h pretreatment with ATRA/FK228 prior to doxorubicin significantly reduced its cytotoxicity.

Next, the effects of ATRA alone or in combination with verapamil on ABCB1 protein expression and transport function in vincristine-resistant mouse lymphocytic leukemia cells (L1210/VCR) were assessed [78]. Verapamil, a well-known ABCB1 inhibitor, was shown to suppress ABCB1 expression and transport function. When combined with ATRA, the suppression of ABCB1 and function was significantly more pronounced than with verapamil alone. Interestingly, ATRA alone did not affect ABCB1 expression or transport function in these cells. The study revealed that verapamil enhances ATRA’s effects, not by direct inhibition of ABCB1 but potentially by preventing the cytochrome P450-mediated metabolism of ATRA. This allows ATRA to exert its activity on ABCB1 expression. Importantly, ATRA was not identified as an ABCB1 substrate since the differences in radiolabeled ATRA uptake were not observed between ABCB1-positive and negative cells, even in the presence of verapamil. The study demonstrated that combining the two agents may provide a greater benefit in inhibiting the transporter than using either one alone.

Similarly, in a subsequent study on L1210 cells, ATRA demonstrated a significantly inhibitory effect on ABCB1 activity, along with a reduction in *ABCB1* mRNA and protein levels, but only when combined with verapamil [80]. Moreover, the effect varied depending on whether ABCB1 expression was constitutively upregulated or introduced via transfection. Inhibition on efflux activity was observed only in cells with naturally vincristine-induced ABCB1 overexpression (L1210/R), whereas no effect was detected in cells with overexpression induced by transfection via plasmid DNA (L1210/T). 9-*Cis*-retinoic acid, a geometric isomer of ATRA, had no significant effect on changes in ABCB1 efflux activity in both cell lines, either as monotherapy or in combination with verapamil, despite the observed upregulation of *ABCB1* mRNA and protein levels. The authors suggest that the inhibitory effect of ATRA on ABCB1 in L1210 cells occurs because of transcriptional regulation of *ABCB1* following the activation of RARs. RARs and RXRs are members of the nuclear superfamily. They function as ligand-activated transcription factors that bind directly to DNA and regulate the expression of target genes. Unlike 9-*cis*-retinoic acid, which is an agonist of both RARs and RXRs, ATRA is a selective agonist of RARs. However, also selective RXRs agonist, bexarotene, showed inhibitory effects on ABC transporters [72]. Surprisingly, despite the lack of effect of retinoic acid stereoisomers on ABCB1 efflux activity in monotherapy, these compounds enhanced the inhibitory effect of vincristine on cell viability in both cell lines overexpressing the respective transporter. This observation suggests that mechanisms other than efflux inhibition may contribute to the compound’s ability to combat drug resistance.

In doxorubicin-resistant human T-cell lymphoma cells (CEM/ADR5000), ATRA outperformed verapamil in suppressing ABCB1 activity at non-cytotoxic concentrations of 20, 50, and 100 μM in the Rh-123 assay, and at 10, 20, 50, and 100 μM in the calcein-AM assay [79]. These concentrations were below the IC_50_ value (157.60 ± 8.33) for the respective cell line, as determined by the methylthiazoltetrazolium (MTT) assay.

In summary, only two retinoids, ATRA and 9-*cis*-retinoic acid, have been evaluated for their impact on ABC transporters (exclusively ABCB1) in hematological malignancies, most commonly in leukemic cell models. The observed outcomes varied depending on several key factors, including the cell type, the chemical properties of the compound, the baseline level of transporter expression or activity, the sequence of retinoid administration, the nature of transporter expression (endogenous versus ectopic), and whether the compounds were used in combination with verapamil. However, the precise mechanisms by which these compounds positively or negatively modulate ABC transporters in leukemias, or lymphoma cells remain poorly elucidated. Importantly, inhibition of drug efflux by retinoid was noted even at sub-cytotoxic concentrations, underscoring the potential of these compounds to restore chemosensitivity without inducing significant toxicity.

### 3.2. Retinoids and ABC Transporters in Gastrointestinal Tumors

Gastrointestinal tumors frequently exhibit elevated expression of ABC transporters [122,123] contributing drug resistance against established and investigational anticancer agents [124,125,126]. Therefore in vitro investigations into the effects of retinoids on these proteins were performed. However, during the period covered by this study, such experiments were conducted exclusively using cell lines derived from colorectal carcinomas.

For example, the effects of ATRA and its synthetic derivative, 6-OH-11-O-hydroxyphenanthrene (IIF, patent WIPO W000/117143), were examined on doxorubicin-resistant colorectal cancer cells (LoVo/MDR) [84]. Both compounds demonstrated dose- and time-dependent inhibition of cell growth in MTT colorimetric and clonogenic assays, with IIF showing significantly greater efficacy than ATRA in MDR cells. Additionally, both retinoids showed pro-apoptotic activity, with IIF inducing a higher level of apoptosis in resistant cells compared to ATRA. However, DNA fragmentation, a hallmark of late-stage apoptosis, was observed only in IIF-treated LoVo/MDR cells, further supporting its stronger pro-apoptotic effect. Regarding ABCB1 modulation, both IIF and ATRA notably reduced ABCB1 protein levels in LoVo/MDR cells. Overall, the study indicates that IIF outperformed ATRA in inhibiting cell proliferation and promoting apoptosis, alongside a clear downregulation of ABCB1 protein expression in MDR colon cancer cells. These findings underscore the superior therapeutic potential of IIF and suggest that synthetic retinoids like IIF may offer a promising strategy for overcoming MDR in colorectal cancer. The effect of the compound on ABC transporter activity was not assessed in this study.

Other researchers investigated the therapeutic potential of retinol as a modulator of *ABCB1* gene expression in human colorectal cancer cell lines with varying *ABCB1* expression levels: HT29 (very low *ABCB1* expression) and SW620 (high *ABCB1* expression) [85]. Interestingly, retinol demonstrated more pronounced antiproliferative effects in cells that overexpress *ABCB1*. A notable reduction in SW620 cell growth was recorded at retinol concentrations as low as 20 μM compared to 40 μM for HT29 cells. Furthermore, retinol at non-cytotoxic dose (7 μM) significantly decreased *ABCB1* gene expression in SW620 cells. The simultaneous administration of retinol and the antioxidant mannitol reversed this effect, indicating a redox-dependent mechanism underlying *ABCB1* downregulation. In contrast, retinol treatment did not affect *ABCB1* expression in HT29 cells. Additionally, a 24 h pretreatment with a non-cytotoxic dose of retinol significantly enhanced the sensitivity of SW620 cells to etoposide after 72 h of incubation, as evidenced by a lower IC_50_ in pretreated cells compared to those not pretreated (0.20 ± 0.07 μM vs. 0.64 ± 0.12 μM). This positive effect was negated by mannitol, a reactive oxygen species (ROS) scavenger. Since SW620 cells express elevated levels of ABCB1, and retinol inhibited its expression via a redox mechanism, this finding suggests that retinol pre-treatment enhances SW620 cell susceptibility to etoposide by reducing ABCB1-mediated drug efflux, thereby increasing etoposide efficacy.

Additionally, retinoids were not shown to be substrates for ABCB1 [78,127], and there were no notable changes in the IC_50_ values for etoposide in HT29 cells that do not express *ABCB1*. This implies that the increased cytoxicity of etoposide in retinol-pre-treated cells was not a result of substrate competition, but instead by the redox-mediated inhibition of *ABCB1* expression [85]. An earlier scientific study also reported that retinol at a concentration of 7 μM increased the levels of ROS in non-cancer rat Sertoli cells [89]. Moreover, this concentration suppressed the expression of the *mdr1* gene (the rodent equivalent of human *ABCB1*) and the *mdr3* gene, while the *mdr2* gene remained unaffected. The effects of oxidative stress on ABCB1 expression and activity have been recently well characterized [20]. Increased oxidative burden differentially regulates ABCB1 expression and activity. While non-damaging levels of prooxidants (eustress) enhance ABCB1 expression and function, excessive levels (distress) suppress them. Several factors have been identified that enhance the expression or activity of ABCB1, including hypoxia-inducible factor 1-alpha (HIF-1α), NF-κB, CAR, PXR and nuclear factor erythroid 2-related factor 2 (Nrf2). Additionally, oxidative stress can alter the integrity of the plasma membrane, potentially affecting the function of membrane-associated proteins.

In human colorectal carcinoma Caco-2 cells, ATRA showed a dose-dependent inhibition of ABCB1 efflux activity, despite having no detectable effect on *ABCB1* mRNA expression [79]. Furthermore, the compound increased doxorubicin accumulation in these cells in tested concentrations (1–250 μM). The IC_50_ value for ATRA in this cell line was 102.30 ± 10.91 μM, indicating that the concentrations at which ABCB1 efflux was inhibited were also within the sub-cytotoxic range. Equally important is the finding that ATRA increased chemosensitivity to etoposide, vinblastine, cisplatin, 5-fluorouracil, and doxorubicin (listed in order of effectiveness) in Caco-2 cells, with the interactions being synergistic. In contrast, antagonistic effect was observed with paclitaxel. Antagonism was also noted when combined with the antifungal agent amphotericin B. With the antimicrobial agent cycloheximide, an additive effect was observed.

The effects of ATRA molecule and its two synthetic derivatives, EC19 (*meta*-isomer, 3-(5,5,8,8-tetramethyl-5,6,7,8-tetrahydronaphthalen-2-ylethynyl)benzoic acid) and EC23 (*para*-isomer, 4-(5,5,8,8-tetramethyl-5,6,7,8-tetrahydronaphthalen-2-ylethynyl)benzoic acid), on the gene expression of *ABCB1*, *ABCC1*, and *ABCG2*, as well as on the protein expression of ABCB1 and ABCC1 in Caco-2 cells, were investigated in a recent study [71]. The researchers also assessed their influence on ATPase activity. These compounds differ in their affinity for RARs [128]: EC19 is a selective RAR-β agonist, while EC23 acts as a pan-agonist for RAR-α, RAR-β, and RAR-γ. ATRA also activates all three RAR isoforms, though with lower potency than EC23. In addition to inducing apoptosis, necrosis, and genotoxicity, retinoids exhibited significant inhibitory effects on selected ABC transporters, though the effects varied between substances. ATRA significantly downregulated *ABCC1* and *ABCG2* mRNA but did not significantly alter the protein expression of ABCB1 or ABCC1. In contrast, compound EC19 significantly suppressed both gene and protein expression of ABCB1 and ABCC1. Compound EC23 reduced *ABCC1* and *ABCG2* gene expression and decreased ABCB1 and ABCC1 protein levels. Despite both ATRA and EC23 acting as pan-RAR agonists, their divergent effects on protein expression suggest involvement of additional regulatory mechanisms in ABC transporter modulation. Furthermore, both synthetic EC-retinoids significantly reduced calcium-independent ATPase activity, unlike ATRA, which did not inhibit the protein expression of the ABC transporters studied, potentially explaining this discrepancy. Consistent with these observations, EC19 and EC23 acted synergistically with both RARβ2-selective agonist (AC261066) and RARγ-selective agonist (CD437), whereas ATRA did not show such interaction.

On the other hand, other researchers highlighted a potential link between ABCG2 upregulation and the chemo-preventive effect of ATRA [64]. They reported an increase in *ABCG2* mRNA after 8 h of incubation and a rise in protein expression after 2 days in Caco-2 cells exposed to ATRA at concentrations between 0.01 and 25 µM, relative to the control. A significant upregulation of *ABCG2* mRNA was also observed following treatment with 1 µM of the compound at 6, 12, and 24 h. Furthermore, agonistic activation of RAR (Am580) and, to a greater extent, RXR (CD2608) resulted in an upregulation of *ABCG2* expression. An elevation in protein expression was evident as well, most prominently after RXR activation. The greatest upregulation of expression was detected upon combined treatment with both agonists. Additionally, the study demonstrated a clear dose-dependent enhancement in the efflux of a phase II metabolite, B[*a*]P-3-sulfate, originating from the pre-carcinogenic food contaminant benzo[*a*]pyrene, which is a known substrate of ABCG2. However, a key limitation is that the study evaluated efflux in cancer cells, not in normal healthy cells.

These investigations demonstrated that retinoids may inhibit ABC transporters at the gene or protein level, suppress transporter activity, or enhance chemosensitivity to conventional cytostatics or investigational compounds, with outcomes varying according to compound type, cell line, dose, or basal transporter expression. Notably, these effects were mostly observed at sub-cytotoxic concentrations. Furthermore, development of synthetic derivatives was prompted by one promising compound demonstrating superior properties. Nevertheless, thorough validation of these effects and their underlying mechanisms is still limited. Added to this, in vivo studies are notably absent, and there is a significant lack of research involving non-colorectal gastrointestinal cancer models. It is also crucial to further explore the potential chemo-preventive effects of retinoids, particularly in healthy cells.

### 3.3. Retinoids and ABC Transporters in Malignancies of the Urogenital Tract

Research on retinoids targeting ABC transporters in urogenital tract tumors remains extremely limited, with only two studies published in the period under investigation.

In tumor cells with high CD44 expression, isolated from human breast cancer cell lines MDA-MB-231 and MDA-MB-468, ATRA did not have a significant effect on ABCB1 protein expression after 7 days incubation [88].

The impact of ATRA on ABC transporters was also examined in human primary ovarian cell lines derived from patient samples [65]. These cells exhibited high levels of ABCB1 (paclitaxel-resistant cells, W1PR) or ABCG2 (topotecan-resistant cells, W1TR), along with detectable levels of aldehyde dehydrogenase 1 family member A1 (ALDH1A1) in both W1PR and W1TR cells. Interestingly, an overexpression of ALDH1 enzymes is linked to the development of drug resistance in many malignancies [129,130,131] including some types of ovarian cancer [132,133]. The inhibition of ALDH1 isoenzymes has also been highlighted as significant in the context of CSCs [134]. Furthermore, it has been reported that ALDH1A1 contributes to enhanced DNA repair and a poly ADP-ribose polymerase inhibitor (PARPi) resistance in ovarian cancer cells by inducing ATRA production and activating its associated signaling pathway [135]. On the other hand, a time-dependent reduction in ABCB1 protein expression was observed in W1PR cells (days 2, 3, and 4) treated by ATRA, while a decrease in ABCG2 was noted in W1TR (days 3 and 4), which corresponded with a decline in ALDH1A1 expression in both lines [65]. This enzyme is also known to catalyze the conversion of retinol into ATRA. Based on this, it is possible that ATRA inhibited ALDH1 gene expression through a feedback mechanism. Notably, mRNA levels did not align with protein expression levels. ATRA alone did not impact the survival of these cell lines; however, when used as a 48 h pretreatment, it significantly improved the effectiveness of the subsequently administered chemotherapeutic agents (paclitaxel and topotecan) after 72 h. A recent study showed that ATRA also increased responsiveness to the targeted therapy niraparib, a PARPi, in both in vitro and in vivo settings [131]. However, the effect on ABC transporters was not investigated in this study.

These findings suggest that ATRA may be a promising agent for reducing drug efflux-mediated chemoresistance in ovarian cancer, as it demonstrated dual inhibition of two distinct ABC transporters. Independent studies have confirmed high expression of ABCB1 in paclitaxel-resistant ovarian cancer cells and ABCG2 in topotecan-resistant cells [136,137]. Additionally, elevated ABCG2 expressions are characteristic of ovarian CSCs [138]. Furthermore, the timing of ATRA administration, particularly pretreatment, appears to play a critical role in treatment outcomes of ovarian cancer. However, the precise mechanisms by which ATRA inhibits ABC transporters remain only partially understood and require further investigation. Future research should aim to elucidate additional regulatory factors influencing ABC transporter expression. For example, in ovarian cancer cells, ABCB1 expression may be regulated by transcription factors such as forkhead box protein P1 (FOXP1) [139], or through hormonal modulation by estrogen and progesterone [140]. Of equal importance, other ABC transporters beyond ABCB1 may also be overexpressed in ovarian cancer cells and contribute to chemoresistance [141].

### 3.4. Retinoids and ABC Transporters in Other Cells or Cell Models

Tarapcsák et al. 2017 [68] investigated the effects of retinol, 13-*cis*-retinoic acid, and retinyl acetate on ABC transporters and demonstrated that they inhibit the activity of ABCB1 and ABCG2 in vitro, using cell line models overexpressing the respective efflux transporters (mouse fibroblast cell line overexpressing ABCB1, NIH 3T3 MDR1 and Madine-Darby canine kidney cell line overexpressing ABCG2, MDCK ABCG2). This inhibition led to increased accumulation of the corresponding fluorescent substrates, calcein and mitoxantrone, respectively. The inhibitory effect of retinoids on the transporters was further supported by their ability to suppress substrate-stimulated ATPase activity. Additionally, retinyl acetate was also identified as a substrate of ABCB1; however, this did not affect its cytotoxicity, likely due to compensatory passive influx of the vitamin. These retinoids also modified membrane properties, increasing rigidity and density. Such changes may influence the ability of substrates to enter the membrane and reach efflux transporters. Interestingly, stereoisomers of 13-*cis*-retinoic acid, including 9-*cis*-retinoic acid and ATRA, along with other vitamin A derivatives such as retinyl palmitate and retinyl propionate, did not exhibit these effects. The researchers suggest that these differences may stem from distinct membrane-binding sites or localization patterns of the individual derivatives. Although these findings highlight a novel role for retinoids in modulating ABC transporter function, their physiological relevance is limited by their low (nanomolar) endogenous concentrations compared to the micromolar concentrations required for significant inhibition. However, therapeutic dosing or supplementation may raise local levels sufficiently to inhibit efflux transporters (e.g., in the intestine), potentially altering drug absorption or distribution within the intestine and at blood-organ barriers [68]. Moreover, effective concentrations may vary depending on the type of retinoid. For example, retinol can reach micromolar levels even under physiological conditions [142].

In CSCs derived from spheroids of human A375 melanoma cells, treatment with a combination of ATRA and resveratrol resulted in reduced *ABCG2* mRNA levels and increased sensitivity to docetaxel [73]. The authors also noted elevated SRY-box transcription factor 9 (SOX9) expression and reduced SOX10 levels. However, it remains unclear from this study whether these changes are directly responsible for the observed decrease in *ABCG2* expression. In contrast, other researchers have reported that SOX9 knockdown leads to downregulation of ABCG2 in hepatocellular carcinoma [143].

In brief, this study confirmed the efficacy of certain retinoids in inhibiting more than one ABC transporters and evaluated their interactions not only with the transporter proteins themselves but also with the cellular membrane. Moreover, retinoids may inhibit ABC transporters in CSCs.

## 4. Rexinoids and ABC Transporters

Rexinoids possess partial structural similarity to traditional retinoids, yet they are chemically distinct entities that selectively interact with RXRs, in contrast to retinoids, which primarily activate RARs [144,145].

The only representative used in practice is bexarotene approved by the Food and Drug Administration (FDA) for refractory cutaneous T-cell lymphoma [146]. In addition to its antiproliferative activity [147,148], it has demonstrated chemosensitizing properties, enhancing the effectiveness of paclitaxel and gemcitabine [149,150] in lung cancer; cisplatin, doxorubicin, and paclitaxel in prostate cancer [151], and paclitaxel, cisplatin and doxorubicin in breast cancer [63,152].

Within the group of rexinoids, bexarotene was the only compound examined for its influence on the gene expression of *ABCB1* and other ABC transporters, as well as its role in mediating chemoresistance.

Table 2 outlines the specific effects of rexinoid (bexarotene) on ABC transporters, with its chemical structure depicted in Figure 2 and Figure S2.

The effects of bexarotene on ABC transporters were studied in human pluripotent embryonal carcinoma NT2 (NTera2/D1) cells, a model characterized by CSC-like properties [72]. Bexarotene treatment promoted cellular differentiation, suppressed expression of stemness-related genes and CSC-associated markers, and significantly downregulated the expression of key ABC transporter genes (*ABCB1*, *ABCC1*, *ABCC2* and *ABCG2*). In contrast, treatment with cisplatin resulted in upregulation of these genes. However, co-administration of cisplatin with non-cytotoxic dose of bexarotene led to a pronounced suppression of ABC transporter gene expression, indicating a potential chemosensitizing effect of rexinoid. This reversal of cisplatin-induced chemoresistance by bexarotene was further supported by a reduction in the size and number of tumor spheroids derived from NT2 cells. Mechanistically, the compound downregulated ABC transporter gene expression by upregulating regulatory factor X1 (RFX1), which directly binds to the promoters of these genes and represses their transcription. On the other hand, Nrf2 acted as a transcriptional activator of *ABCB1*, *ABCC1*, and *ABCG2* (but not *ABCC2*), while bexarotene was identified as an inhibitor of Nrf2 activity. Interestingly, molecular docking aided in the identification of potential regulators of ABC genes in this study. RFX1 is a pleiotropic transcription factor, frequently downregulated in various cancers, with demonstrated tumor-suppressive functions including inhibition of cellular proliferation, modulation of immune responses, induction of apoptosis, and mitigation of chemoresistance [153]. Nrf2 is a regulatory factor involved in the transcription of several antioxidant enzymes and in the control of inflammation [154]. Oxidative stress triggers the activation of Nrf2 by causing the dissociation of its repressor, Keap1, from the Keap1–Nrf2 complex. Nrf2 then moves into the nucleus, where it binds to antioxidant response elements (AREs) and stimulates the expression of protective enzymes and transporter proteins [20]. Previous studies have already demonstrated its capacity to suppress MDR genes in tumour cells [155]. Furthermore, modulation of both positive (Nrf2) and negative (RFX1) regulators of ABC genes by bexarotene was facilitated via the retinoid RXRα signaling [72]. Bexarotene also demonstrated a significant inhibitory effect on HIF-1α in NT2 cells, both independently and in combination with cisplatin [72]. Recently, it was demonstrated that HIF-1α may contribute to the control of ABC transporter expression [20,156].

Other researchers reported that the combination of paclitaxel and bexarotene prevented the development of paclitaxel resistance in MDA-MB-231 cells (*ABCB1*+, *ABCB11*−, *ABCC1*−, *ABCC2*−, *ABCC3*−, *ABCG2*−), likely through downregulation of *ABCB1* mRNA expression and a reduction in calcein-AM efflux [63]. Furthermore, co-administration of paclitaxel and bexarotene resulted in a greater reduction in *ABCB1* mRNA expression and efflux transporter activity compared to sequential administration.

Overall, this study confirmed the potential of a novel class of agents in inhibiting ABC transporters present in CSCs and cancer cells, thereby reducing drug resistance. Furthermore, it provided insights into the possible mechanisms underlying these effects. Interestingly, this drug may also help prevent the development of chemoresistance.

## 5. Carotenoids and ABC Transporters

Carotenoids are a large family of biologically active, mostly colored, lipophilic tetraterpenoids [157] that share chemical similarities with retinoids (Figure 2), as both are isoprenoid derivatives containing conjugated double bonds [158]. There are several hundred carotenoids with different chemical structures [159] and certain carotenoids, such as β-carotene, α-carotene, and β-cryptoxanthin, also serve as dietary precursors of retinoids.

Many carotenoids are naturally found in fruits, vegetables, plants, fungi, algae, and photosynthetic bacteria, but can also be found in food of animal origin (dairy goods, eggs, fish, cow and horse liver) and cereals [157,160]. Additionally, they are used as natural colorants [161]. Since humans are unable to synthesize carotenoids endogenously, they are obtained through diet or nutritional supplements [162].

Chemically, they are classified as carotenes (e.g., α-carotene, β-carotene, and lycopene), which are hydrocarbon molecules that may have cyclic structures at one or both ends; and xanthophylls (e.g., fucoxanthin, lutein, neoxanthin, violaxanthin, and zeaxanthin), which are oxygen-containing derivatives of carotenes (Figure S3) [55,161,163,164]. Carotenes are soluble in organic solvents and insoluble in polar solvents, while xanthophylls are soluble in both polar solvents and organic solvents [161]. In addition, apocarotenoids (including, e.g., crocetin and crocin) represent a distinct group of carotenoids produced by the enzyme-mediated oxidative breakdown of carotenes or xanthophylls [165]. In nature, most carotenoids exist in the all-(*E*) geometric configuration. However, (*Z*)-isomerization may provide several physicochemical advantages (e.g., improved solubility) or biological benefits (e.g., greater bioavailability and higher antioxidant capacity) [166].

In the context of cancer, these compounds exhibit a broad range of anticancer activities, including inhibition of proliferation, angiogenesis, and metastasis; modulation of apoptosis, necroptosis, and autophagy; induction of cell differentiation, immune modulation, anti-inflammation, antioxidant and pro-oxidant effects, enhancement of gap functional communication, as well as modulation of nuclear receptor superfamily, growth factors, and Wnt/β-catenin signaling [50,165,167].

The ability of carotenoids to reverse MDR has also been described [79,165]. However, the conflicting results from the previously mentioned CARET study should also be considered [102]. In the same line, a recent meta-analysis did not support the role of β-carotene in chemoprevention and instead highlighted an increased lung cancer risk associated with its use, particularly among smokers [168]. A more recent meta-analysis suggested that the chemo-preventive effects of carotenoids depend on both the cancer type and the dosage used [58].

The individual effects of carotenoids on ABC transporters are detailed in Table 3, and their corresponding chemical structures are illustrated in Figure S3.

### 5.1. Carotenoids and ABC Transporters in Hematologic Malignancies

Like retinoids, carotenoids have been studied in selected cell lines of hematologic malignancies (primarily lymphoma) exhibiting MDR phenotype in vitro.

Molnár et al. 2004 [81] investigated the effect of 12 natural carotenoids (antheraxanthin, capsanthin, capsorubin, lutein, lycopene, lycophyll, violaxanthin, zeaxanthin, α-carotene, β-carotene, α-cryptoxanthin and β-cryptoxanthin) isolated from several plants (Table 3) on the efflux activity in mouse T-cell lymphoma cells transfected by the human *ABCB1* gene (L5178Y–MDR1/A) using the Rh-123 assay [81]. The observed effect depended on the chemical structure of carotenoid molecule: three compounds (capsanthin, capsorubin and lycophyll) showed strong efflux inhibition (a fluorescence activity ratio, FAR, higher than 20 at a concentration of 20 µg/mL), seven compounds (antheraxanthin, lutein, lycopene, violaxanthin, zeaxanthin, α-cryptoxanthin and β-cryptoxanthin) exhibited moderate inhibition (a FAR higher than 2 at a concentration of 20 µg/mL), and two carotenoids (α-carotene and β-carotene) had no effect on efflux (a FAR below 1 at both 2 µg/mL and 20 µg/mL concentrations) in the studied cell line. Some carotenoids (antheraxanthin, capsanthin, capsorubin, lutein, zeaxanthin, and β-cryptoxanthin) increased Rh-123 accumulation at concentrations as low as 2 µg/mL. Thus, anti-efflux activity was demonstrated by linear, non-cyclic compounds (lycopene and lycophyll) as well as by compounds bearing hydroxyl (antheraxanthin, lutein, zeaxanthin, α-cryptoxanthin and β-cryptoxanthin), epoxide (antheraxanthin and violaxanthin), or keto functionality (capsanthin and capsorubin). On the other hand, α-carotene and β-carotene are pure hydrocarbons lacking oxygen atoms and are also classified as nonpolar compounds. Furthermore, nonpolar carotenoids have been found to exhibit a lower propensity for membrane interaction compared to their polar counterparts [169], which may account for the limited impact of carotenes on efflux inhibition. However, lycopene, despite being nonpolar, could enhance Rh-123 accumulation.

Additional carotenoids were tested on the same cell line (L5178Y–MDR1/A), which exhibits confirmed ABCB1 expression as verified by immunocytochemistry, at concentrations of 4 and 40 µg/mL [82]. Most of the tested compounds ((5*R*,8*R*)-capsochrome, (5*R*,8*S*)-capsochrome, (5*S*,8*R*)-capsochrome, (5*S*,8*S*)-capsochrome, (8′*R*)-luteoxanthin, (8′*S*)-luteoxanthin, monoepoxy-β-carotene, (9′*Z*)-neoxanthin, (9*Z*)-violaxanthin, (9*Z*)-zeaxanthin, and (13*Z*)-zeaxanthin) or their combinations (chrysanthemaxanthin + flavoxanthin and (13*Z*)-lutein + (13′*Z*)-lutein) exhibited strong accumulation of Rh-123 (a FAR higher than 20 at a concentration of 40 µg/mL). The highest FAR values (exceeding 50) were observed with compounds (5*R*,8*R*)-capsochrome, (5*S*,8*R*)-capsochrome, (8′*R*)-luteoxanthin, (8′*S*)-luteoxanthin, (9′*Z*)-neoxanthin, and (9*Z*)-violaxanthin. Other carotenoids including aurochrome, diepoxy-β-carotene, luteochrome and mutatochrome exhibited a moderate inhibitory effect on efflux activity (a FAR higher than 2 at a concentration of 20 µg/mL). Except for aurochrome, all previously mentioned carotenoids demonstrated measurable anti-efflux activity at concentrations as low as 4 µg/mL. One potential mechanism by which these compounds inhibit efflux involves their incorporation into the hydrophobic core of the cell membrane [170], resulting in decreased lipid fluidity and permeability which can be associated with multidrug resistance [171]. On the other hand, two carotene derivatives 15,15′-dehydrodiepoxy-β-carotene and monoepoxy-α-carotene did not promote Rh-123 accumulation at any concentration, with a FAR of less than 0.75. The ability of selected carotenoids to modulate resistance to epirubicin, an anthracycline, considered a substrate of ABC transporters, was also evaluated using a combination assay. Compounds (9*Z*)-zeaxanthin, (8′*S*)-luteoxanthin, (5*S*,8*S*)-capsochrome, and (13*Z*)-zeaxanthin (listed in order of effectiveness) demonstrated synergistic effects with the tested anthracycline following 72 h of incubation, while (9*Z*)-violaxanthin and monoepoxy-β-carotene exhibited additive interactions. The most pronounced enhancement of epirubicin efficacy was observed with (9*Z*)-zeaxanthin. The authors identified the presence of a (*Z*)-configuration at the double bond as a critical structural feature contributing to efflux inhibition, as derivatives bearing this configuration demonstrated significantly higher Rh-123 accumulation compared to their counterparts with all-*trans* configuration. However, it should be noted that the studies [81,82] did not employ identical compound concentrations, which may influence the interpretation of these findings. Another key structural feature of the investigated compounds associated with the ability to overcome efflux-mediated resistance was the presence of –OH functional groups at the C-3 and C-3′ positions. Like in the previous study [81], the presence of epoxide groups may also have contributed to this effect. Furthermore, a decline in the efficacy of the compounds was also noted following storage for several months at −20 °C, likely due to oxidative degradation [82]. Generally, exposure to oxygen, light, heat, acids, and metal ions may decrease activity of these compounds [159,161].

Similarly, in another doxorubicin-resistant T-cell lymphoma cell line (CEMADR5000), selected carotenoids (canthaxanthin, crocin, fuoxanthin, and β-carotene) reduced efflux activity at concentrations ranging from 10 to 100 µM, as evidenced by increased intracellular accumulation of Rh-123 and calcein [79]. Notably, efflux inhibition was observed at concentrations below the IC_50_ values of the individual compounds.

To sum up, both naturally derived carotenoids and their modified derivatives demonstrated effective inhibition of efflux activity in lymphoma cells at non-cytotoxic concentrations, depending on chemical structure and cell line. This activity was particularly pronounced among compounds featuring hydroxyl, epoxide, or keto functional groups, as well as those displaying a (*Z*)-configuration at their double bond. Notably, like retinoids [68], the carotenoids also interacted with the cellular membrane, suggesting membrane-related mechanisms may contribute to their inhibitory action.

### 5.2. Carotenoids and ABC Transporters in Gastrointestinal Malignancies

During the period under review, the ability of carotenoids to interact with ABC transporters in gastrointestinal malignancies was investigated in colorectal, hepatocellular, and gastric carcinoma cell lines.

Several carotenoids (antheraxanthin, fetoxanthin, lutein, luteoxanthin, neoxanthin, violaxanthin, violeoxanthin and β-cryptoxanthin) were evaluated for their ability to inhibit efflux activity in colon cancer Colo 320 cell line expressing both ABCB1 and lung resistance protein (LRP) [83]. All tested compounds, except for neoxanthin, significantly reduced Rh-123 efflux at a concentration of 4 µg/mL; neoxanthin showed inhibitory activity only at a higher concentration of 40 µg/mL. Four compounds (antheraxanthin, fetoxanthin, lutein, and violeoxanthin) exhibited FAR values above 5, with compound violeoxanthin being the most effective, reaching a FAR value of 13.45 at 40 µg/mL. The tested concentrations did not induce significant apoptosis.

A subsequent study on Caco-2 colon cancer cells demonstrated a dose-dependent increase in the accumulation of Rh-123 and calcein in response to selected carotenoids (canthaxanthin, crocin, fucoxanthin, and β-carotene) at concentrations ranging from 1 to 250 µM [79]. However, the fluorescence intensity of individual compounds depended not only on their chemical properties but also on the type of assay employed. As the cytotoxic IC_50_ values for all tested carotenoids in the given cell line were above 120 μM, their efflux-inhibitory effects, likely involving ABCB1, were also observed at non-cytotoxic concentrations. In addition, all compounds at concentration of 40 μM increased doxorubicin accumulation confirmed by increasing fluorescence intensity. Upon 24 h co-incubation with conventional chemotherapeutic agents (cisplatin, doxorubicin, etoposide, 5-fluorouracil, paclitaxel, and vinblastine), canthaxanthin and fucoxanthin exhibited exclusively synergistic interactions. The greatest degree of synergy was recorded for 5-fluorouracil, reflected by combination index values of 0.47 and 0.58, respectively. β-carotene also exhibited synergistic interactions with the evaluated cytostatic agents, except for paclitaxel, with which an antagonistic effect was observed. Crocin demonstrated the lowest efficacy in reversing chemoresistance, exhibiting antagonistic interactions with paclitaxel, 5-fluorouracil, and etoposide. In contrast, it showed synergistic effects when combined with the other chemotherapeutic agents (cisplatin, doxorubicin, and vinblastine). Consistent with previous studies, the most effective carotenoids in this investigation were those containing hydroxyl and epoxide functional groups (fucoxanthin) or keto groups (canthaxanthin). The authors further hypothesized that the presence of the epoxide functional group may contribute to the alkylation of efflux transporters. In addition, RT-PCR analysis demonstrated that treatment with fucoxanthin, crocin, canthaxanthin, and β-carotene (listed in order of effectiveness) at a concentration of 40 µM for 48 h resulted in a reduction of ABCB1 mRNA expression levels in these cancer cells, which points to another factor responsible for ABCB1 suppression.

Fucoxanthin, a marine non-pro-vitamin A carotenoid naturally found in edible brown algae [172], also showed a substantial, dose-dependent reduction in both basal and rifampicin-stimulated *ABCB1* mRNA expression in colorectal adenocarcinoma LS174T cells, beginning at a concentration of 5 µM [46]. Importantly, rifampicin is known to induce both cytochrome P450 enzymes [173] and ABCB1 [174].

Furthermore, fucoxanthin demonstrated similar properties in the human hepatocellular carcinoma cell line (HepG-2) as it did in LS174T cells [46]. The observed inhibition of *ABCB1* mRNA was likely mediated through suppression of PXR signaling, as a dose-dependent reduction in the interaction between PXR and its coactivator, Steroid Receptor Coactivator-1 (SRC-1), was detected. Additionally, the study reported inhibition of human CAR (hCAR) signaling. Interestingly, PXR and hCAR are transcription factors that regulate the expression of transporter and metabolic genes in response to xenobiotic stress [18,175,176].

The same compound exhibited strong anti-efflux activity in doxorubicin-resistant HepG-2 (HepG-2/Dox) [70]. Combination treatment with commercially prepared fucoxanthin and doxorubicin enhanced doxorubicin accumulation and cytotoxicity in a dose-dependent manner. Notably, following 24 h incubation, this carotenoid at a concentration of 20 µM significantly reduced the IC_50_ value of doxorubicin from 13.77 ± 1.29 to 2.19 ± 0.19 µM, with the interaction classified as synergistic. Fucoxanthin also significantly inhibited Rh-123 efflux at a non-cytotoxic concentration of 20 µM in cancer cells, exhibiting a greater effect than verapamil. Moreover, fluorescence intensity was additionally amplified upon co-administration of fucoxanthin with doxorubicin. Equally important, the tested carotenoid exhibited lower cytotoxicity in the resistant cell line (HepG-2/Dox) relative to the parental line (HepG-2), implying potential recognition and extrusion by efflux transporters.

Next, crocin was evaluated in both parental and doxorubicin-resistant gastric cancer cell lines (EPG85-257 and EPG85-257RDB), where it did not affect *ABCB1* mRNA expression in either cell line. However, it enhanced sensitivity to doxorubicin starting at a concentration of 25 µM. Encouragingly, the antiproliferative IC_50_ values for this carotenoid consistently remained above 75 µM in both cell lines across all time points [86].

Research showed that various carotenoids from all chemical classes may exert chemosensitizing effects in cells derived from the gastrointestinal system. These effects may be attributed to their ability to inhibit drug efflux, reduce *ABCB1* mRNA expression, or potentially alkylate proteins, although the latter property was not assessed in the present studies. One report identified inhibition of the PXR and hCAR signaling pathways by carotenoids as a potential mechanism for *ABCB1* suppression. In some cases, the underlying cause remained undetermined. Overall, the activity of carotenoids at non-toxic concentrations further underscores their therapeutic promise. Surprisingly, in contrast to L5178Y–MDR1/A cell line [81], β-carotene demonstrated anti-efflux activity in colorectal cancer cells [79].

### 5.3. Carotenoids and ABC Transporters in Malignancies of the Urogenital Tract

In addition to hematologic and gastrointestinal models, the effects of carotenoids on ABC transporters or transporter-mediated chemoresistance have also been investigated in cancer cell lines derived from gynecological malignancies, primarily including breast, ovarian, and cervical carcinomas.

Despite expectations, a broad spectrum of naturally occurring carotenoids extracted from paprika (capsanthin, capsorubin, α-cryptoxanthin and β-cryptoxanthin) and other plants (antheraxanthin, lutein, lycopene, lycophyll, violaxanthin, zeaxanthin, α-carotene, β-carotene) showed no inhibitory effect on Rh-123 accumulation in human breast carcinoma cells (MDA-MB-231) when tested at concentrations of 2 and 20 µg/mL [81]. This finding could be attributed to the likely absence of MDR-associated transporter expression in the cell line used, as the fluorescence signal following Rh-123 administration was comparable to that of the control.

Subsequently, capsanthin and zeaxanthin were evaluated at higher concentration (40 µg/mL) in drug-resistant sublines of another human breast cancer cell line MCF-7 [87]. These included sublines resistant to docetaxel (MCF-7/Doc), doxorubicin (MCF-7/Dox), paclitaxel (MCF-7/Pac), and vincristine (MCF-7/Vinc). Both carotenoids enhanced Rh-123 accumulation, with FAR values exceeding 3 across all resistant sublines. Zeaxanthin demonstrated superior anti-efflux activity, with FAR values consistently above 5 in all sublines, whereas FAR values for capsanthin remained below 5. Only zeaxanthin showed a higher accumulation effect on Rh-123 accumulation than verapamil. Furthermore, zeaxanthin exhibited greater efficacy in combination treatments with conventional chemotherapeutics, that are well-established substrates of ABCB1. Specifically, it showed a synergistic interaction with doxorubicin after 72 h of incubation, and additive interactions with paclitaxel, vincristine, and docetaxel (listed in order of effectiveness). However, capsanthin demonstrated only additive effects with doxorubicin, paclitaxel, and docetaxel, while its interaction with vincristine was classified as indifferent.

The anti-efflux potential of the broad panel of carotenoids was also assessed in a doxorubicin-resistant human breast cancer cell line (MCF-7/Dox), in parallel with the previous evaluation performed in mouse lymphoma L5178Y cells [82]. Most of the tested compounds ((5*R*,8*R*)-capsochrome, (5*R*,8*S*)-capsochrome, (5*S*,8*R*)-capsochrome, (5*S*,8*S*)-capsochrome, diepoxy-β-carotene, (13*Z*)-lutein + (13′*Z*)-lutein), (8′*R*)-luteoxanthin, luteochrome, (8′*S*)-luteoxanthin, monoepoxy-β-carotene, mutatochrome, (9*Z*)-violaxanthin, (9*Z*)-zeaxanthin, and (13*Z*)-zeaxanthin) induced increased Rh-123 accumulation at the tested concentrations (4 and 40 µg/mL); however, the FAR values observed in the breast cancer cells were markedly lower compared to those obtained in the L5178Y cell line. The highest FAR value in breast cancer cells was 2.37, observed for compound monoepoxy-β-carotene, whereas some compounds ((5*R*,8*R*)-capsochrome, (5*S*,8*R*)-capsochrome, (8′*R*)-luteoxanthin, (8′*S*)-luteoxanthin, (9′*Z*)-neoxanthin, and (9*Z*)-violaxanthin) in the L5178Y cells exhibited FAR values exceeding 50. Overall, the FAR values of tested carotenoids were lower than that of verapamil (FAR = 11.9) in doxorubicin-resistant subline MCF-7 cells. Despite the lower FAR values, carotenoids (9*Z*)-violaxanthin and (13*Z*)-zeaxanthin (listed in order of effectiveness) demonstrated a synergistic interaction with epirubicin in this cell line, while compounds (9*Z*)-zeaxanthin and (8′*S*)-luteoxanthin exhibited an additive effect. In contrast, although compound (5*S*,8*S*)-capsochrome increased Rh-123 accumulation, it exhibited antagonism in combination with epirubicin, indicating that additional resistance mechanism beyond enhanced efflux may be involved in these cells. Combination of chrysanthemaxanthin and flavoxanthin only slightly enhanced efflux inhibition in the breast cancer cells (FAR = 1.1 at concentration 40 µg/mL). Compounds aurochrome, 15,15′-dehydrodiepoxy-β-carotene, monoepoxy-α-carotene and (9′*Z*)-neoxanthin were associated with decreased efflux, with FAR values ranging from 0.62 to 0.90. The differential effects of the same carotenoids across various cell lines may be attributed to variations in their interactions with the lipid membrane. It is well established that cell lines differ in their lipid composition [171], and polar compounds, typically more effective in modulating efflux, have been shown to incorporate more readily into membrane regions enriched in unsaturated lipids [177].

The highly potent fucoxanthin was also evaluated in the same cell line (MCF-7/Dox) [70]. It significantly increased Rh-123 accumulation in a dose-dependent manner, and this effect was further amplified in the presence of doxorubicin, as evidenced by a marked increase in fluorescence intensity. A synergistic interaction between a non-cytotoxic dose of the examined carotenoid (20 µM) and doxorubicin was observed, resulting in a substantial reduction in the IC_50_ value of doxorubicin (from 18.71 ± 1.91 to 2.22 ± 0.21 µM). A similar pattern of all observed effects of fucoxanthin was noted in the doxorubicin-resistant human ovarian adenocarcinoma (SKOV-3/Dox) cells. The synergistic interaction between the carotenoid and doxorubicin resulted in a reduction of doxorubicin’s IC_50_ from 16.77 ± 1.81 μM to 3.68 ± 0.32 µM. Moreover, fucoxanthin significantly downregulated mRNA expression of the three major ABC transporters (*ABCB1*, *ABCC1*, and *ABCG2*) in MCF-7/Dox cells. The most pronounced suppression was noted for *ABCB1* (change fold = 0.11), while the least reduction was observed for *ABCG2* (fold change = 0.44). Interestingly, doxorubicin alone also reduced the expression of these transporters, and the combination treatment (fucoxanthin and doxorubicin) produced the greatest decrease in mRNA levels across all three ABC transporters. Additionally, in the tested cell line, treatment with fucoxanthin, doxorubicin, and their combination resulted in decreased mRNA expression of *PXR* gene [70], a known regulator of several ABC transporters [19]. Moreover, PXR is a crucial transcription factor in the biotransformation of xenobiotic drugs, regulating the gene expression of selected phase I (mainly CYP3A4), II (mainly GST), and III metabolic enzymes (mainly ABCT) [178]. Fucoxanthin was also shown to modulate ABCB1 expression through activation of the *PXR* signaling pathway [46].

A previous study examining crocin derived from saffron (*Crocus sativus* L.) plant in cisplatin-resistant ovarian cancer cells (A2780/RCIS) demonstrated a strong inhibitory effect on the mRNA expression of *ABCC1* and *ABCC2* [66]. The compound also significantly enhanced sensitivity to doxorubicin, a known substrate of MRP [179] at doses lower than those needed to inhibit cell proliferation [66]. Notably, the A2780/RCIS cell line differed from its parental counterpart (A2780) primarily by its markedly elevated *ABCC2* expression. While the compound also reduced doxorubicin resistance in the parental line, the authors did not assess *ABCC1* and *ABCC2* mRNA expression levels in that line.

Crocetin encapsulated in poly(lactic-*co*-glycolic acid) (PLGA) nanoparticles (PLGA-Crt NPs; particle size: 239.8 ± 9 nm) was also tested on the same ovarian cancer cell lines and demonstrated superior antiproliferative activity compared to crocetin alone [69]. This formulation significantly inhibited *ABCC2* mRNA, but not *ABCC1* mRNA, in A2780/RCIS cells. Across all tested concentrations (25–200 µM), it inhibited doxorubicin efflux both directly and, more notably, indirectly in a dose-dependent manner, with effects starting at 25 µM. The IC_50_ was determined to be 96.0 ± 2 µM.

In vincristine-resistant human cervical carcinoma (KB-vin) cells, characterized by high *ABCB1* mRNA expression and minimal expression of *ABCC1* and *ABCG2*, β-carotene (100 µM) significantly upregulated *ABCB1* mRNA levels [67]. However, it markedly enhanced chemosensitivity to paclitaxel and doxorubicin at non-cytotoxic concentration (50 µM). A modest enhancement in 5-fluorouracil efficacy was also observed, whereas the cytotoxic activity of etoposide was attenuated in the presence of this carotenoid. In contrast, β-carotene did not significantly enhance chemosensitivity in human cervical carcinoma (HeLaS3) cell line lacking substantial expression of all three ABC transporters.

Overall, most of the tested carotenoids, including nanoparticle form, effectively reversed chemoresistance in gynecological malignancy-derived cell lines expressing ABC transporters. They enhanced chemosensitivity to anthracyclines (doxorubicin and epirubicin), taxanes (paclitaxel and docetaxel), and, to a lesser extent, vinca alkaloids (vincristine), depending on the compound and cell line. The underlying mechanisms ranged from reduced efflux activity and downregulation of *ABCB1*, *ABCC1*, *ABCC2* and *ABCG2* expression depending on the compound, as seen in breast and ovarian cancer cells, whereas in cervical cancer cells, the effects were independent of ABC transporter-mediated efflux inhibition. Notably, one study also highlighted a potential mechanism by which carotenoids may regulate ABC transporter expression via PXR.

### 5.4. Carotenoids and ABC Transporters in Other Malignancies or Other Cell Models

In mitoxantrone-resistant human non-small-cell lung carcinoma (NCI-H460/MX20) cells exhibiting elevated mRNA expression of the *ABCG2* and *ABCB1* compared to the parental line (NCI-H460), β-carotene at a non-cytotoxic concentration (50 µM) significantly enhanced sensitivity to mitoxantrone [67].

The influence of β-carotene on ABC transporters was further examined by the same researchers [67] using three engineered cell models derived from Flp-In™-293 human embryonic kidney cells, each stably expressing one of the transporter genes: *ABCB1* (*ABCB1*/Flp-In™-293), *ABCC1* (*ABCC1*/Flp-In™-293), or *ABCG2* (*ABCG2*/Flp-In™-293). Protein expression and activity of each transporter was confirmed in all models, with Multidrug Activity Factor (MAF) values for ABCB1, ABCC1, and ABCG2 substantially surpassing the 25% cut-off, validating their activity. In the calcein-AM assay, the compound notably increased calcein accumulation in *ABCB1*-overexpressing cells (*ABCB1*/Flp-In™-293), while no such effect was observed in *ABCC1*-expressing cells (*ABCC1*/Flp-In™-293), suggesting selective inhibition of ABCB1. This selective inhibition was further supported by ATPase activity measurements, where the compound elevated both basal and verapamil-induced ATPase activity, indicating interaction with the ABCB1 transporter. Furthermore, in the ABCB1 model, the compound enhanced the intracellular retention of Rh-123 and doxorubicin, leading to a significant increase in the cytotoxicity of doxorubicin. Notably, this functional inhibition occurred without altering *ABCB1* mRNA levels, implying a non-transcriptional mechanism of action. In addition, a minor structural modification of ABCB1 protein was observed following treatment with the carotenoid. Regarding ABCG2, mitoxantrone accumulation assay demonstrated only modest inhibitory effect. Similarly, findings from the eFluxx-ID^®^ Green Dye assay (ENZO Life Sciences, Inc. Farmingdale, NY, USA) confirmed the compound’s limited impact on ABCG2 and lack of inhibition of ABCC1, reinforcing its selective inhibition of ABCB1 activity.

In contrast to the results obtained in the study on the L5178Y–MDR1/A cell line [81], but consistent with observation on Caco-2 cell line [79], β-carotene demonstrated anti-efflux activity in NCI-H460 and *ABCB1*/Flp-In™-293 cells [67]. In the transfected cell model, this effect was shown to be both selective for ABCB1 and independent of changes in gene expression. Furthermore, the compound acted as a chemosensitizer with selected traditional cytostatics. Interestingly, the work of Teng et al. 2016 [67] employed a more comprehensive approach to evaluating compound activity on ABC transporters.

## 6. Summary of Potential Mechanisms Involved in Regulation of ABC Transporters

To date, only a limited number of studies have explored the potential mechanisms by which vitamin A-based molecules affect the expression and activity of ABC transporters. A summary of these mechanisms is presented in Figure 5.

Details of these mechanisms are discussed in the relevant sections of the manuscript; this section provides only a short summary of the key findings.

Oxidative stress is likely involved in the regulation of ABC transporters. It may influence transporter function directly by altering membrane properties such as fluidity and density, or indirectly by modulating the transcription of ABC transporter genes via HIF-1α. Generally, vitamin A-based molecules have been shown to both increase and decrease oxidative stress [56]. Several retinoids (13-*cis*-retinoic acid, retinol and retinyl acetate) discussed in this article were reported to reduce membrane fluidity and density, which was accompanied by decreased efflux activity of ABCB1 and ABCG2 [68]. Additionally, the compounds can be directly incorporated into the membrane, resulting in changes to its properties [170,171]. In the case of retinol, increased oxidative stress was observed alongside reduced ABCB1 expression [85]. Furthermore, bexarotene decreased HIF-1α levels and downregulated the expression of *ABCB1*, *ABCC1*, *ABCC2*, and *ABCG2* transporter genes [72].

Vitamin A-based molecules may also suppress the transcription of ABC transporter genes by inhibiting signaling molecules such as PXR, hCAR, SRC-1, and Nrf2 [46,72], or by upregulating RFX1 expression [72]. Conversely, another study reported that ATRA enhanced ABCB1 expression and activity by promoting the recruitment of NF-YA to the *ABCB1* promoter region [77].

The role of ALDH1A1 in regulating the expression or function of ABC transporters remains poorly understood. It is unclear whether the ATRA-induced reduction in ALDH1A1 protein levels is a coincidental finding or directly contributes to the observed decrease in ABCB1 protein expression [65].

## 7. Conclusions and Future Directions

Vitamin A-based molecules are widely recognized for their chemo-preventive and anticancer properties. This study identifies modulation of efflux transporter activity as another potentially important property of these compounds. Several members of this group, including retinoids, rexinoid, and carotenoids, have demonstrated notable potential to inhibit the expression or activity of ABCB1, a key efflux transporter implicated in MDR in both hematological and solid tumors. Some compounds have also shown the capacity to inhibit other members of the ABC transporter family, suggesting their potential as dual or multimodal modulators of efflux transport. Given the overlapping substrate specificities among ABC transporters, further studies are warranted to investigate the effects of these compounds on multiple transporters concurrently. Furthermore, considering the existence of multiple isoforms of ABC transporters, investigating compound-specific effects on individual transporter subtypes represents a valuable direction for future research. Comparative studies examining the anti-efflux and chemosensitizing properties of vitamin A-based molecules versus other lipophilic vitamins may also be valuable.

Notably, several of the molecules studied also exhibited chemosensitizing effects when combined with conventional cytotoxic agents. As many newer targeted therapies are also substrates of ABC transporters, it is essential to evaluate potential interactions between these agents and vitamin A-based molecules.

Importantly, these compounds may themselves act as substrates of ABC transporters. Therefore, a comprehensive understanding of their transporter-specific interactions is necessary, potentially aided by in silico approaches such as molecular docking.

A particularly advantageous feature of vitamin A-based molecules is their ability to inhibit ABC transporters at non-cytotoxic concentrations. Their activity against ABC transporters expressed in CSCs also represents a promising therapeutic benefit. However, the observed effects of these compounds vary depending on multiple factors, including molecular structure, chemical modifications, concentration, duration of exposure, treatment schedule, cell type, mode of transporter overexpression, and co-administration with other agents. In some cases, paradoxical effects have been reported, such as transporter activation or reduced chemosensitivity. These inconsistencies require further mechanistic elucidation.

Furthermore, a comprehensive summary of the effects of vitamin A-based molecules on bacterial transporters could provide valuable insights, given the structural and functional analogies between human and bacterial transport systems.

Despite promising in vitro findings, a major limitation of current knowledge lies in the lack of in vivo studies evaluating the effects of these compounds on ABC transporters. Most data have been generated using cancer cell lines or engineered cells overexpressing ABC transporters. Advanced preclinical systems such as 3D tumor spheroids, organoids, co-culture systems, or organ-on-a-chip platforms may serve as valuable intermediates before conducting in vivo and clinical investigations. In vivo validation is critical, particularly because ABC transporters are involved not only in drug efflux of chemotherapeutics but also in the transport of endogenous molecules and a wide range of pharmaceuticals. Notably, achieving effective control over xenobiotic efflux in healthy tissues without compromising drug accumulation in tumor cells continues to pose a significant challenge. Moreover, non-efflux roles of ABC transporters are increasingly recognized and should be considered. Another notable limitation of current studies is the insufficient methodological diversity used to assess transporter modulation. A comprehensive approach incorporating gene expression, protein levels, and functional activity is essential, as these parameters do not always correlate directly.

Given the large number of structurally diverse vitamin A-based molecules, particularly carotenoids, it will be necessary to prioritize the most promising candidates. This process can be supported by in silico tools and artificial intelligence (e.g., machine learning models), which may also facilitate the design of novel analogues with improved selectivity, efficacy, and safety profiles. Interestingly, synthetic analogues have already demonstrated superior tolerability, selectivity, potency, and reduced toxicity compared to their natural counterparts. Similarly, vitamin A-based molecules produced by microbial sources may offer additional therapeutic advantages.

Furthermore, ongoing research is essential to elucidate the mechanisms by which these substances modulate transporter activity, and to inform the development of personalized, transporter-targeted therapeutic strategies.

Finally, the low bioavailability of some vitamin A-based compounds presents an additional hurdle. To overcome this, various pharmaceutical strategies, such as encapsulation in liposomes, micelles, or nanogels, may be necessary to enhance their delivery and therapeutic efficacy.

To conclude, although certain vitamin A-based molecules are already employed in clinical settings, there is not enough evidence to support their effectiveness in overcoming MDR driven by increased efflux through ABC transporters. Their potential in this regard is promising, but well-designed, long-term in vivo and clinical studies are necessary to validate their use for this specific purpose.

## Figures and Tables

**Table 1:** Overview of the effects of retinoids studied on ABC transporters.

Drug	Drug Origin	Cell Line/Organism	Effects on ABC Transporters and Chemosensitivity (Drug Concentration; Incubation Time)	Molecular Mechanism	Ref.
All-*trans*-retinoic acid (ATRA, Tretinoin)	U	AML cells from human individuals	Variable effect on *ABCB1* mRNA and ABCB1 substrate accumulation (0.1 μM; 72 h)	Possible inverse effect of *Egr1* mRNA on *ABCB1* expression or positive effect on ABCB1 substrate accumulation in some cells and some incubation periods; no regulatory effect of *WT1*	[75]
	U	AML cells from human individuals	↑ABCB1 substrate accumulation (45 mg/m^2^/d; 72 h prior to the standard treatment)	U	[74]
	C	Caco-2	↑*ABCG2* mRNA (0.01, 0.1, 1, 10 and 25 μM; 8 h), (1 μM; 6, 12 and 24 h); ↑ABCG2 protein expression (0.01, 0.1, 1, 10 and 25 μM; 2 days), ↑efflux of B[a]P-3-sulfate (0.1, 1, 5 and 10 μM; 48 h)	RAR/RXR signaling	[64]
	C	Caco-2	↑ABCB1 substrate accumulation (1–250 µM; 30 min); synergism with cisplatin, DOX, ETO, 5-FU and VINB, antagonism with PAC (40 μM; 24 h); no decrease in *ABCB1* mRNA (40 μM; 48 h)	U	[79]
	C	Caco-2	↓mRNA of *ABCC1* and *ABCG2*, no change in *ABCB1* mRNA (IC_50_ = 97.70 ± 9.0, 24 h); ↓protein expression of ABCB1 and ABCC1 (IC_50_, 24 h); no impact on calcium-independent ATPase and no synergism with AC261066 and CD437 (IC_50_; 24 h)	U	[71]
	C	CEM/ADR5000	↑ABCB1 substrate accumulation (10, 20, 50 and 100 µM; 30 min pretreatment)	U	[79]
	C	H9	↑*ABCB1* mRNA, no impact on ABCB1 substrate accumulation (5 μM; 48 h)	U	[76]
	C	H9/RAR	↑*ABCB1* mRNA; ↓ABCB1 substrate accumulation (5 μM; 48 h)	U	[76]
	C	Kasumi-1, Kasumi-6	↑*ABCB1* mRNA (1 μM; U) alone and in combination with FK228	U	[77]
	U	Kasumi-1	↑*ABCB1* mRNA and ↓ABCB1 substrate accumulation (0.1 μM; 1–72 h)	No regulatory effect of *Egr1* and *WT1*	[75]
	C	KG-1	↑*ABCB1* mRNA; ↓ABCB1 substrate accumulation (5 μM; 48 h)	U	[76]
	U	KG-1	Maintenance of basal *ABCB1* mRNA and ABCB1 substrate accumulation (0.1 μM; 1–72 h)	No regulatory effect of *Egr1* and *WT1*	[75]
	C	KG-1/RAR	↑*ABCB1* mRNA; ↓ABCB1 substrate accumulation (5 μM; 48 h)	U	[76]
	C	K562	↑*ABCB1* mRNA; ↓ABCB1 substrate accumulation (5 μM; 48 h)	U	[76]
	C	K562/RAR	↑*ABCB1* mRNA; ↓ABCB1 substrate accumulation (5 μM; 48 h)	U	[76]
	C	LoVo/MDR	↓ABCB1 protein expression (20 μM; 48 h)	U	[84]
	U	L1210/S	No significant change in *ABCB1* mRNA and protein expression (3.3 μM; U)	Different effects in particular cells, probably altered gene transcription	[80]
	U	L1210/R	No effect in monotherapy, in combination with VER: *↓ABCB1* mRNA and protein expression, ↑ABCB1 substrate accumulation, ↑chemosensitivity to VINC (3.3 μM; U)	Different effects in particular cells; probably altered gene transcription	[80]
	U	L1210/T	↑*ABCB1* mRNA and protein expression in monotherapy and with VER, no effect on ABCB1 substrate accumulation, ↑chemosensitivity to VINC (3.3 μM; U)	Different effects in particular cells; probably altered gene transcription	[80]
	C	L1210/VCR	No effect in monotherapy, in combination with VER: ↓protein expression and ↑ABCB1 substrate accumulation (3.3 μM; 20 h)	Likely through VER-mediated CYP450 inhibition of retinoid metabolism	[78]
	C	MDA-MB-231, MDA-MB-468	No significant change in ABCB1 protein expression (5 μM; 7 days)	U	[88]
	C	MDCK ABCG2	No effect on ABCG2 substrate accumulation (10, 25, 50, 100 μM; 20 min)	No effect on membrane density/fluidity	[68]
	C	MM cancer stem-like cells	↓*ABCG2* mRNA (20 μM; 24 h) and ↑chemosensitivity to DOC when ATRA (20 μM; 24 h + 24 h) used with RES	U	[73]
	C	NB4	↑*ABCB1* mRNA; undetermined impact on efflux activity (5 μM; U)	U	[76]
	C	NB4	↑*ABCB1* mRNA and protein expression (1 μM; 72 h), ↑ABCB1 substrate efflux (1 μM; U), especially in combination with FK228; ↓cytotoxicity of DOX in ATRA/FK228 pretreatment (1 μM; 24 h); ↑cytotoxicity of DOX in ATRA/FK228 posttreatment (1 μM; 48 h)	↑ H4 and H3-Lys9 acetylation, recruitment of NF-YA to the CCAAT box in the ABCB1 promoter	[77]
	C	NB4/RAR	↑*ABCB1* mRNA; undetermined impact on efflux activity (5 μM; U)	U	[76]
	C	NIH 3T3 MDR1	No effect on ABCB1 substrate accumulation (10, 25, 50 μM; 20 min)	No effect on membrane properties	[68]
	U	W1PR	No change in *ABCB1* mRNA (5 μM; 1–4 d); ↓ABCB1 protein expression (5 μM; 1–4 days); ↑chemosensitivity to paclitaxel (5 μM; 48 + 72 h)	↓ALDH1A1 protein expression	[65]
	U	W1TR	Transiently ↑*ABCG2* mRNA (5 μM; 1–2 d); ↓ABCG2 protein expression (5 μM; 3–4 d); ↑chemosensitivity to topotecan (5 μM; 48 + 72 h)	↓ALDH1A1 protein expression	[65]
9-*Cis*-retinoic acid (Alitretinoin)	U	L1210/S	No significant change in *ABCB1* mRNA and protein expression (3.3 μM; U)	Different effects in particular cells, probably altered gene transcription	[80]
	U	L1210/R	↑*ABCB1* mRNA in monotherapy and with VER, ↑ABCB1 protein expression in combination with VER, no effect on ABCB1 substrate accumulation, ↑vincristine chemosensitivity (3.3 μM; U)	Different effects in particular cells, probably altered gene transcription	[80]
	U	L1210/T	↑*ABCB1* mRNA in combination with VER, ↑ABCB1 protein level in monotherapy and with VER, no effect on ABCB1 substrate accumulation, ↑vincristine chemosensitivity (3.3 μM; U)	Different effects in particular cells, probably altered gene transcription	[80]
	C	MDCK ABCG2	No effect on ABCG2 substrate accumulation (10, 25, 50, 100 μM; 20 min)	No effect on membrane properties	[68]
	C	NIH 3T3 MDR1	No effect on ABCB1 substrate accumulation (10, 25, 50 μM; 20 min)	No effect on membrane properties	[68]
13-*Cis*-retinoic acid (Isotretinoin)	C	MDCK ABCG2	↑ABCG2 substrate accumulation (10–100 μM; 20 min)	Alteration of membrane properties	[68]
	C	NIH 3T3 MDR1	↑ABCB1 substrate accumulation (25 μM; 20 min)	Alteration of membrane fluidity and density	[68]
EC19	C	Caco-2	↓mRNA of *ABCB1* and *ABCC1*, no change in *ABCG2* mRNA (IC_50_ = 27.20 ± 1.8; 24 h); ↓protein expression of ABCB1 and ABCC1 (IC_50_; 24 h); ↓activity of calcium-independent ATPase and synergism with AC261066 and CD437 (IC_50_; 24 h)	U	[71]
EC23	C	Caco-2	↓mRNA of *ABCC1* and *ABCG2*, no change in *ABCB1* mRNA (IC_50_ = 23.00 ± 1.2, 24 h); ↓protein expression of ABCB1 and ABCC1 (IC_50_, 24 h); ↓activity of calcium-independent ATPase and synergism with AC261066 and CD437 (IC_50_; 24 h)	U	[71]
IIF	Synthetic	LoVo/MDR	↓ABCB1 protein expression (20 μM; 48 h)	U	[84]
Retinol (vitamin A)	U	HT29	No effect on *ABCB1* mRNA expression (7 μM; 24 h)	U	[85]
	C	MDCK ABCG2	↑ABCG2 substrate accumulation (50–100 μM; 20 min)	Alteration of membrane fluidity and density	[68]
	C	NIH 3T3 MDR1	↑ABCB1 substrate accumulation (50 μM; 20 min)	Alteration of membrane fluidity and density	[68]
	U	SW620	↓*ABCB1* mRNA (7 μM; 24 h) and ↑chemosensitivity to etoposide after pretreatment (7 μM; 24 h)	↑oxidative state	[85]
Retinyl acetate	C	MDCK ABCG2	↑ABCG2 substrate accumulation (100 μM; 20 min)	Alteration of membrane fluidity and density	[68]
	C	NIH 3T3 MDR1	↑ABCB1 substrate accumulation (50 μM; 20 min)	Alteration of membrane fluidity and density	[68]
Retinyl palmitate	C	MDCK ABCG2	No effect on ABCG2 substrate accumulation (10, 25, 50, 100 μM; 20 min)	No effect on membrane properties	[68]
	C	NIH 3T3 MDR1	No effect on ABCB1 substrate accumulation (10, 25, 50 μM; 20 min)	No effect on membrane properties	[68]
Retinyl propionate	C	MDCK ABCG2	No effect on ABCG2 substrate accumulation (10, 25, 50, 100 μM; 20 min)	No effect on membrane properties	[68]
	C	NIH 3T3 MDR1	No effect on ABCB1 substrate accumulation (10, 25, 50 μM; 20 min)	No effect on membrane properties	[68]

ALDH1A1, aldehyde dehydrogenase 1 family member 1A; C, commercial; CYP450, cytochrome P450; d, days; DOC, docetaxel; DOX, doxorubicin; *Egr1*, early growth response 1 gene; ETO, etoposide; h, hours; min, minutes; PAC, paclitaxel; RAR, Retinoic acid receptor; Ref., reference; RES, resveratrol; RXR, retinoid X receptor; U, undetermined; VER, verapamil; VINB, vinblastine; VINC, vincristine; *WT1*, Wilms’ tumor suppressor gene; 5-FU; 5-fluorouracil. The designations of cell lines are provided in the Abbreviations section. The symbols ↑ and ↓ indicate increased and decreased levels.

**Table 2:** Overview of the effects of rexinoid studied on ABC transporters.

Drug	Drug Origin	Cell Line/Organism	Effects on ABC Transporters and Chemosensitivity (Drug Concentration; Incubation Time)	Molecular Mechanism	Ref.
Bexarotene	C	MDA-MB-231 (resistant variants)	↓*ABCB1* mRNA and ↑ABCB1 substrate accumulation in PAC-resistant cells (1 µM, U); ↑chemosensitivity to CIS, DOX, and PAC (1 µM, 1–90 days)	U	[63]
	U	NT2	↓mRNA of *ABCB1*, *ABCC1*, *ABCC2*, and *ABCG2* (25 µM; U); ↑chemosensitivity to CIS (10 µM; 48 h)	↑RXRα signaling → ↑RFX1; ↓Nrf2; ↓HIF-1α	[72]

C, commercial; CIS, cisplatin; DOX, doxorubicin; HIF-1α, hypoxia-inducible factor 1α; Nrf2, nuclear factor erythroid 2-related factor 2; PAC, paclitaxel; Ref., reference; RFX1, regulatory factor X1; RXR, retinoid X receptor; U, undetermined. The designations of cell lines are provided in the Abbreviations section. The symbols ↑ and ↓ indicate increased and decreased levels.

**Table 3:** Overview of the effects of carotenoids studied on ABC transporters.

Drug	Drug Origin	Cell Line/Organism	Effects on ABC Transporters and Chemosensitivity (Drug Concentration; Incubation Time)	Molecular Mechanism	Ref.
Antheraxanthin	U	Colo 320	↑ABCB1 substrate accumulation (4/40 µg/mL; 10 min)	U	[83]
	*Viola tricolor*, yellow flowers	L5178Y (MDR1/A)	↑ABCB1 substrate accumulation (2/20 µg/mL; 10 min)	U	[81]
Aurochrome	Lab internal collection	L5178Y (MDR1/A)	↑ABCB1 substrate accumulation (40 µg/mL; 10 min)	U	[82]
	Lab internal collection	MCF-7 (DOX-resistant)	Slightly ↓ABCB1 substrate accumulation (4/40 µg/mL; 10 min)	U	[82]
Canthaxanthin	C	Caco-2	↑ABCB1 substrate accumulation (1–250 µM; 30 min); synergistic effect with CIS, DOX, ETO, 5-FU, PAC, and VINB (40 µM; 24 h); ↓*ABCB1* mRNA (40 µM; 48 h)	U	[79]
	C	CEM/ADR5000	↑ABCB1 substrate accumulation (10, 20, 50 and 100 µM; 90 min)	U	[79]
Capsanthin	*Capsicum annuum*, red paprika	L5178Y (MDR1/A)	↑ABCB1 substrate accumulation (2/20 µg/mL; 10 min)	U	[81]
	U	MCF-7/Doc	↑ABCB1 substrate accumulation (40 µg/mL; 10 min); additive effect with DOC (U; 72 h)	U	[87]
	U	MCF-7/Dox	↑ABCB1 substrate accumulation (40 µg/mL; 10 min); additive effect with DOX (U; 72 h)	U	[87]
	U	MCF-7/Pac	↑ABCB1 substrate accumulation (40 µg/mL; 10 min); additive effect with PAC (U; 72 h)	U	[87]
	U	MCF-7/Vinc	↑ABCB1 substrate accumulation (40 µg/mL; 10 min); indifferent effect with VINC (U; 72 h)	U	[87]
Capsorubin	*Capsicum annuum*, red paprika	L5178Y (MDR1/A)	↑ABCB1 substrate accumulation (2/20 µg/mL; 10 min)	U	[81]
(5*R*,8*R*)-Capsochrome	Lab internal collection	L5178Y (MDR1/A)	↑ABCB1 substrate accumulation (4/40 µg/mL; 10 min)	U	[82]
	Lab internal collection	MCF-7 (DOX-resistant)	↑ABCB1 substrate accumulation (4/40 µg/mL; 10 min)	U	[82]
(5*R*,8*S*)-Capsochrome	Lab internal collection	L5178Y (MDR1/A)	↑ABCB1 substrate accumulation (4/40 µg/mL; 10 min)	U	[82]
	Lab internal collection	MCF-7 (DOX-resistant)	↑ABCB1 substrate accumulation (4/40 µg/mL; 10 min)	U	[82]
(5*S*,8*R*)-Capsochrome	Lab internal collection	L5178Y (MDR1/A)	↑ABCB1 substrate accumulation (4/40 µg/mL; 10 min)	U	[82]
	Lab internal collection	MCF-7 (DOX-resistant)	↑ABCB1 substrate accumulation (4/40 µg/mL; 10 min)	U	[82]
(5*S*,8*S*)-Capsochrome	Lab internal collection	L5178Y (MDR1/A)	↑ABCB1 substrate accumulation (4/40 µg/mL; 10 min); synergism with EPI (U; 72 h)	U	[82]
	Lab internal collection	MCF-7 (DOX-resistant)	↑ABCB1 substrate accumulation (4/40 µg/mL; 10 min); antagonism with EPI (U; 72 h)	U	[82]
Chrysanthemaxanthin + flavoxanthin	Lab internal collection	L5178Y (MDR1/A)	↑ABCB1 substrate accumulation (4/40 µg/mL; 10 min)	U	[82]
	Lab internal collection	MCF-7 (DOX-resistant)	↑ABCB1 substrate accumulation (40 µg/mL; 10 min)	U	[82]
Crocetin nanoparticles (PLGA-Crt NPs)	*Crocus sativus* L., saffron	A2780/RCIS	↓*ABCC2* mRNA and no decrease in *ABCC1* mRNA (25, 50, 100 and 200 µM; 48 h); ↓efflux of DOX (25, 50, 100 and 200 µM; 48 h)	U	[69]
Crocin	*Crocus sativus* L., saffron	A2780/RCIS	↓*ABCC1* mRNA (25 and 100 µM; 48 h) and *ABCC2* mRNA (25, 50 and 100 µM; 48 h); ↑chemosensitivity to DOX (25, 50, and 100 µM; 24, 48 and 72 h)	U	[66]
	C	Caco-2	↑ABCB1 substrate accumulation (1–250 µM; 30 min); synergistic effect with CIS, DOX, and VINB (40 µM; 24 h), antagonism with ETO, 5-FU and PAC (40 µM; 24 h); ↓*ABCB1* mRNA (40 µM; 48 h)	U	[79]
	C	CEM/ADR5000	↑ABCB1 substrate accumulation (10, 20, 50 and 100 µM; 30 min)	U	[79]
	*Crocus sativus* L., saffron	EPG85-257	No decrease in *ABCB1* mRNA (25, 50, and 100 µM; 48 h); ↑chemosensitivity to DOX (25, 50, and 100 µM; 24, 48, and 72 h)	U	[86]
	*Crocus sativus* L., saffron	EPG85-257RDB	No decrease in *ABCB1* mRNA (25, 50, and 100 µM; 48 h); ↑chemosensitivity to DOX (25, 50, and 100 µM; 24, 48, and 72 h)	U	[86]
15,15′-Dehydrodiepoxy-β-carotene	Lab internal collection	L5178Y (MDR1/A)	↓ABCB1 substrate accumulation (4/40 µg/mL; 10 min)	U	[82]
	Lab internal collection	MCF-7 (DOX-resistant)	↓ABCB1 substrate accumulation (4/40 µg/mL; 10 min)	U	[82]
Diepoxy-β-carotene	Lab internal collection	L5178Y (MDR1/A)	↑ABCB1 substrate accumulation (4/40 µg/mL; 10 min)	U	[82]
	Lab internal collection	MCF-7 (DOX-resistant)	↑ABCB1 substrate accumulation (4/40 µg/mL; 10 min)	U	[82]
Fetoxanthin	Isolated from apple peel	Colo 320	↑ABCB1 substrate accumulation (4/40 µg/mL; 10 min)	U	[83]
Fucoxanthin	C	Caco-2	↑ABCB1 substrates accumulation (1–250 µM; 30 min); synergism with CIS, DOX, ETO, 5-FU, PAC, and VINB (40 µM; 24 h), ↓*ABCB1* mRNA (40 µM; 48 h)	U	[79]
	C	CEM/ADR5000	↑ABCB1 substrates accumulation (10, 20, 50 and 100 µM; 90 min)	U	[79]
	*Undaria pinnatifida*, wakame	HepG-2	↓*ABCB1* mRNA (1, 5, and 10 µM; 24 h)	↓PXR signaling via inhibition of interaction with SRC-1 coactivator and ↓hCAR	[46]
	C	HepG-2/Dox	↑ABCB1 substrate accumulation (20 µM; 24 h); ↑DOX accumulation and synergism with DOX (20 µM; 30 min)	U	[70]
	*Undaria pinnatifida*, wakame	LS174T	↓*ABCB1* mRNA (5 and 10 µM; 24 h)	↓PXR signaling via inhibition of interaction with SRC-1 coactivator and ↓hCAR	[46]
	C	MCF-7/Dox	↑ABCB1 substrate accumulation (20 µM; 24 h); ↑DOX accumulation and synergism with DOX (20 µM; 30 min); ↓mRNA levels of *ABCB1*, *ABCC1*, and *ABCG2* (U; 24 h)	↓*PXR* mRNA	[70]
	C	SKOV-3/Dox	↑ABCB1 substrate accumulation (20 µM; 24 h); ↑DOX accumulation and synergism with DOX (20 µM; 30 min)	U	[70]
Lutein	U	Colo 320	↑ABCB1 substrate accumulation (4/40 µg/mL; 10 min)	U	[83]
	*Caltha palustris*, marsh marigold	L5178Y (MDR1/A)	↑ABCB1 substrate accumulation (2/20 µg/mL; 10 min)	U	[81]
(13*Z*) + (13′*Z*)-Lutein	Lab internal collection	L5178Y (MDR1/A)	↑ABCB1 substrate accumulation (4/40 µg/mL; 10 min)	U	[82]
	Lab internal collection	MCF-7 (DOX-resistant)	↑ABCB1 substrate accumulation (4/40 µg/mL; 10 min)	U	[82]
Luteochrome	Lab internal collection	L5178Y (MDR1/A)	↑ABCB1 substrate accumulation (4/40 µg/mL; 10 min)	U	[82]
	Lab collection	MCF-7 (DOX-resistant)	↑ABCB1 substrate accumulation (4/40 µg/mL; 10 min)	U	[82]
Luteoxanthin	U	Colo 320	↑ABCB1 substrate accumulation (4/40 µg/mL; 10 min)	U	[83]
(8′*R*)-Luteoxanthin	Lab internal collection	L5178Y (MDR1/A)	↑ABCB1 substrate accumulation (4/40 µg/mL; 10 min)	U	[82]
	Lab internal collection	MCF-7 (DOX-resistant)	↑ABCB1 substrate accumulation (4/40 µg/mL; 10 min)	U	[82]
(8′*S*)-Luteoxanthin	Lab internal collection	L5178Y (MDR1/A)	↑ABCB1 substrate accumulation (4/40 µg/mL; 10 min); synergism with EPI (U; 72 h)	U	[82]
	Lab internal collection	MCF-7 (DOX-resistant)	↑ABCB1 substrate accumulation (4/40 µg/mL; 10 min); additive effect with EPI (U; 72 h)	U	[82]
Lycopene	*Lycopersicon esculentum*, tomato	L5178Y (MDR1/A)	No effect on ABCB1 substrate accumulation (2 µg/mL; 10 min); ↑ABCB1 substrate accumulation (20 µg/mL; 10 min)	U	[81]
Lycophyll	*Solanum dulcamara*, bittersweet nightshade	L5178Y (MDR1/A)	No effect on ABCB1 substrate accumulation (2 µg/mL; 10 min); ↑ABCB1 substrate accumulation (20 µg/mL; 10 min)	U	[81]
Monoepoxy-α-carotene	Lab internal collection	L5178Y (MDR1/A)	↓ABCB1 substrate accumulation (4/40 µg/mL; 10 min)	U	[82]
	Lab internal collection	MCF-7 (DOX-resistant)	↓ABCB1 substrate accumulation (4/40 µg/mL; 10 min)	U	[82]
Monoepoxy-β-carotene	Lab internal collection	L5178Y (MDR1/A)	↑ABCB1 substrate accumulation (4/40 µg/mL; 10 min); additive effect with EPI (U; 72 h)	U	[82]
	Lab internal collection	MCF-7 (DOX-resistant)	↑ABCB1 substrate accumulation (4/40 µg/mL; 10 min); indifferent effect with EPI (U; 72 h)	U	[82]
Mutatochrome	Lab internal collection	L5178Y (MDR1/A)	↑ABCB1 substrate accumulation (4/40 µg/mL; 10 min)	U	[82]
	Lab internal collection	MCF-7 (DOX-resistant)	↑ABCB1 substrate accumulation (4/40 µg/mL; 10 min)	U	[82]
Neoxanthin	U	Colo 320	↑ABCB1 substrate accumulation (40 µg/mL; 10 min)	U	[83]
(9′*Z*)-Neoxanthin	Lab internal collection	L5178Y (MDR1/A)	↑ABCB1 substrate accumulation (4/40 µg/mL; 10 min)	U	[82]
	Lab internal collection	MCF-7 (DOX-resistant)	↓ABCB1 substrate accumulation (4/40 µg/mL; 10 min)	U	[82]
Violaxanthin	U	Colo 320	↑ABCB1 substrate accumulation (4/40 µg/mL; 10 min)	U	[83]
	*Viola tricolor*, yellow flowers	L5178Y (MDR1/A)	↑ABCB1 substrate accumulation (2/20 µg/mL; 10 min)	U	[81]
(9*Z*)-Violaxanthin	Lab internal collection	L5178Y (MDR1/A)	↑ABCB1 substrate accumulation (4/40 µg/mL; 10 min); additive effect with EPI (U; 72 h)	U	[82]
	Lab internal collection	MCF-7 (DOX-resistant)	↑ABCB1 substrate accumulation (4/40 µg/mL; 10 min); synergism with EPI (U; 72 h)	U	[82]
Violeoxanthin	U	Colo 320	↑ABCB1 substrate accumulation (4/40 µg/mL; 10 min)	U	[83]
Zeaxanthin	*Lycium halimifolium*	L5178Y (MDR1/A)	↑ABCB1 substrate accumulation (2/20 µg/mL; 10 min)	U	[81]
	U	MCF-7/Doc	↑ABCB1 substrate accumulation (40 µg/mL; 10 min); additive effect with DOC (U; 72 h)	U	[87]
	U	MCF-7/Dox	↑ABCB1 substrate accumulation (40 µg/mL; 10 min); synergism with DOX (U; 72 h)	U	[87]
	U	MCF-7/Pac	↑ABCB1 substrate accumulation (40 µg/mL; 10 min); additive effect with PAC (U; 72 h)	U	[87]
	U	MCF-7/Vinc	↑ABCB1 substrate accumulation (40 µg/mL; 10 min); additive effect with VINC (U; 72 h)	U	[87]
(9*Z*)-Zeaxanthin	Lab internal collection	L5178Y (MDR1/A)	↑ABCB1 substrate accumulation (4/40 µg/mL; 10 min); synergism with EPI (U; 72 h)	U	[82]
	Lab internal collection	MCF-7 (DOX-resistant)	↑ABCB1 substrate accumulation (4/40 µg/mL; 10 min); additive effect with EPI (U; 72 h)	U	[82]
(13*Z*)-Zeaxanthin	Lab internal collection	L5178Y (MDR1/A)	↑ABCB1 substrate accumulation (4/40 µg/mL; 10 min); synergism with EPI (U; 72 h)	U	[82]
	Lab collection	MCF-7 (DOX-resistant)	↑ABCB1 substrate accumulation (4/40 µg/mL; 10 min); synergism with EPI (U; 72 h)	U	[82]
α-Carotene	*Daucus Carotta*, carrot	L5178Y (MDR1/A)	No effect on ABCB1 substrate accumulation (2/20 µg/mL; 10 min)	U	[81]
β-Carotene	C	*ABCB1*/Flp-In TM-293	↑Calcein accumulation (10, 25, 50, and 100 µM; 30 min); ↑Rh-123 accumulation (IC_50_ = 25.72 ± 0.2 μM; 30 min); ↑DOX accumulation (IC_50_ = 16.81 ± 0.43 μM; 3 h); ↑ABCB1 ATP-ase activity (10–100 μM; U); slight conformational change of ABCB1 protein (100 μM; U); ↑chemosensitivity to DOX (10 and 20 µM; 72 h); no impact on *ABCB1* mRNA (100 µM; 48 h)	U	[67]
	C	*ABCC1*/Flp-In TM-293	No impact on calcein accumulation (10, 25, 50, and 100 µM; 30 min)	U	[67]
	C	*ABCG2*/Flp-In TM-293	↑Mitoxantrone accumulation (10, 25, 50, and 100 µM; 30 min)	U	[67]
	C	Caco-2	↑ABCB1 substrates accumulation (1–250 µM; 30 min); synergism with CIS, DOX, ETO, 5-FU, and VINB (40 µM; 24 h), antagonism with PAC; ↓*ABCB1* mRNA (40 µM; 48 h)	U	[79]
	C	CEM/ADR5000	↑ABCB1 substrates accumulation (10, 20, 50 and 100 µM; 90 min)	U	[79]
	C	KB-vin	↑Chemosensitivity to PAC, DOX and 5-FU, ↓chemosensitivity to ETO (50 µM, 72 h); ↑*ABCB1* mRNA (100 µM; 72 h)	U	[67]
	*Daucus carotta*, carrot	L5178Y (MDR1/A)	No effect on ABCB1 substrate accumulation (2/20 µg/mL; 10 min)	U	[81]
	C	NCI-H460/MX20	↑Chemosensitivity to mitoxantrone (50 µM; 72 h)	U	[67]
α-Cryptoxanthin	Yellow paprika, Valencia orange peels	L5178Y (MDR1/A)	No effect on ABCB1 substrate accumulation (2 µg/mL; 10 min); ↑ABCB1 substrate accumulation (20 µg/mL; 10 min)	U	[81]
β-Cryptoxanthin	U	Colo 320	↑ABCB1 substrate accumulation (4/40 µg/mL; 10 min)	U	[83]
	Yellow paprika, Valencia orange peels	L5178Y (MDR1/A)	↑ABCB1 substrate accumulation (2/20 µg/mL; 10 min)	U	[81]

CIS, cisplatin; C, commercial; hCAR, human constitutive androstane receptor; DOC, docetaxel; DOX, doxorubicin; EPI, epirubicin; ETO, etoposide; PLGA, poly(lactic-*co*-glycolic acid); PLGA-Crt NPs, crocetin encapsulated poly(lactic-*co*-glycolic acid) nanoparticles; PAC, paclitaxel; PXR, pregnane X receptor; Rh-123, Rhodamine 123; SRC-1, steroid receptor coactivator-1; U, unknown or unclear; VINB, vinblastine; VINC, vincristine. The designations of cell lines are provided in the Abbreviations section. The symbols ↑ and ↓ indicate increased and decreased levels.

## Data Availability

The original contributions presented in this study are included in the article and  Supplementary Materials. Further inquiries can be directed to the corresponding authors.

## Supplementary Materials

The following supporting information can be downloaded at: https://www.mdpi.com/article/10.3390/ijms262211157/s1.

## Abbreviations

The following abbreviations are used in this manuscript:

ABC	ATP-binding cassette
ABCB1	ATP-binding cassette subfamily b member 1 (P-glycoprotein)
*ABCB1*/Flp-In TM-293	Flipase recombinase-compatible human embryonic kidney 293 with transfected human *ABCB1*
ABCC1	ATP-binding cassette subfamily c member 1 (multidrug resistance-associated protein 1)
*ABCC1*/Flp-In TM-293	Flipase recombinase-compatible human embryonic kidney 293 with transfected human *ABCC1*
ABCC2	ATP-binding cassette subfamily c member 2 (multidrug resistance-associated protein 2)
ABCC3	ATP-binding cassette subfamily c member 3 (multidrug resistance-associated protein 3)
ABCG2	ATP-binding cassette subfamily g member 2 (breast cancer resistance protein)
*ABCG2*/Flp-In TM-293	Flipase recombinase-compatible human embryonic kidney 293 with transfected human *ABCG2*
ABCT	ATP-binding cassette transporter
AC261066	RARβ-2 agonist
ALDH1	Aldehyde dehydrogenase 1
ALDH1A1	Aldehyde dehydrogenase 1 family member 1A
AML	Acute myeloid leukemia
APL	Acute promyelocytic leukemia
ARE	Antioxidant response element
ATO	Arsenic trioxide
ATP	Adenosine triphosphate
ATPase	Adenosine triphosphatase
ATRA	All-*trans*-retinoic acid
A2780/RCIS	Human ovarian cancer, cisplatin-resistant subline
BCRP	Breast cancer resistance protein
Caco-2	Human colon cancer
CAR	Constitutive androstane receptor
CARET	Carotene and Retinol Efficacy Trial
CD437	RAR-γ selective agonist
CD44	Cluster of Differentiation 44
CEM/ADR5000	Human acute lymphoblastic leukemia (T-cell type), doxorubicin-resistant subline
Colo 320	Human colon cancer overexpressing ABCB1 and lung resistant protein (LRP)
CSC	Cancer stem cell
CYP3A4	Cytochrome P450 3A4
CYP450	Cytochrome P450
EC19	3-(5,5,8,8-tetramethyl-5,6,7,8-tetrahydronaphthalen-2-ylethynyl)benzoic acid
EC23	4-(5,5,8,8-tetramethyl-5,6,7,8-tetrahydronaphthalen-2-ylethynyl)benzoic acid
EPG85-257	Human gastric carcinoma
EPG85-257RDB	Human gastric carcinoma, doxorubicin-resistant subline
Egr1	Early growth response 1 gene
FAB	French-American-British classification system of AML
FAR	Fluorescence activity ratio
FDA	Food and Drug Administration
FK228	Histone deacetylase inhibitor (HDACI), Romidepsin (depsipeptide)
FOXP1	Forkhead box protein P1
5-FU	5-Fluorouracil
GST	Glutathione S-transferase
hCAR	Human constitutive androstane receptor
HeLaS3	Human cervical carcinoma
HepG-2	Human hepatocellular carcinoma
HepG-2/Dox	Human hepatocellular carcinoma, doxorubicin-resistant subline
HIF-1α	Hypoxia-inducible factor 1-alpha
HL-60/RS	Multidrug-resistant human acute promyelocytic leukemia cells overexpressing ABCB1, ABCC1, and ABCG2
HT29	Human colon cancer
H3-Lys9	Histone H3 at lysine 9
H9	Human acute T-cell leukemia
H9/RAR	RARα transfected human acute T-cell leukemia
IC_50_	Half-maximal inhibitory concentration
IIF	6-OH-11-*O*-hydroxyphenanthrene
Kasumi-1	Human acute myeloid leukemia with t(8:21) karyotype
Kasumi-6	Human acute myeloid leukemia
KB-vin	Human cervical carcinoma, vincristine-resistant subline
Keap1	Kelch-like ECH-associated protein 1
KG-1	Human acute myeloid leukemia
KG-1/RAR	RARα transfected human acute myeloid leukemia
K562	Human cell line from the patient in blast crisis of chronic myeloid leukemia
K562/RAR	RARα transfected human cell line from the patient in blast crisis of chronic myeloid leukemia
LoVo/MDR	Human colorectal cancer, doxorubicin-resistant subline
LRP	Lung resistant protein
LS174T	Human colorectal carcinoma
L1210/R	Mouse lymphocytic leukemia, subline with ABCB1-overexpression induced by vincristine
L1210/S	Mouse lymphocytic leukemia/without detectable ABCB1 expression and efflux activity
L1210/T	Mouse lymphocytic leukemia, subline with ABCB1 overexpression induced by transfection
L1210/VCR	Mouse lymphocytic leukemia, vincristine-resistant subline
L5178Y (MDR1/A)	Mouse T-cell lymphoma cells transfected by human *ABCB1* gene
MAF	Multidrug Activity Factor
MCF-7	Human breast cancer
MCF-7/Doc	Human breast cancer, docetaxel-resistant subline
MCF-7/Dox	Human breast cancer, doxorubicin-resistant subline
MCF-7/Pac	Human breast cancer, paclitaxel-resistant subline
MCF-7/Vinc	Human breast cancer, vincristine-resistant subline
MDA-MB-231	Human triple-negative breast cancer
MDA-MB-468	Human triple-negative breast cancer
MDCK ABCG2	Madine-Darby canine kidney cell line overexpressing ABCG2
MDR	Multidrug resistance
MDR1	Multidrug resistance protein 1
mdr1	Multidrug resistance 1 gene (the rodent equivalent of human *ABCB1*)
mdr2	Multidrug resistance 2 gene
mdr3	Multidrug resistance 3 gene
MM CS-like cells	Cancer stem cells derived from spheroids of human A375 melanoma cells
MRP1	Multidrug resistance-associated protein 1 (ABCC1)
MRP2	Multidrug resistance-associated protein 2 (ABCC2)
MRP3	Multidrug resistance-associated protein 3 (ABCC3)
MTT	Methyltiazoltetrazolium assay
MVP	Major vault protein/Lung resistant protein
M1	Acute myeloblastic leukemia without maturation
M2	Acute myeloblastic leukemia with maturation with t(8;21) karyotype
M3	Acute promyelocytic leukemia
M4	Acute myelomonocytic leukemia
NB4	Human acute promyelocytic leukemia
NB4/RAR	RARα transfected human acute promyelocytic leukemia
NCI-H460	Human non-small-cell lung carcinoma
NCI-H460/MX20	Human non-small-cell lung carcinoma, mitoxantrone-resistant subline
NF-YA	Nuclear transcription factor Y alpha
NF-κB	Nuclear factor kappa-B
NIH 3T3 MDR1	Mouse fibroblast cell line overexpressing ABCB1
Nrf2	Nuclear factor erythroid 2-related factor 2
NT2	Human pluripotent embryonal carcinoma
PARPi	Poly ADP-ribose polymerase inhibitor
PI3K/Akt/mTOR	Phosphoinositide 3-kinase/protein kinase B/mechanistic target of rapamycin signaling pathway
PXR	Pregnane X receptor
RAR	Retinoic acid receptor
RFX1	Regulatory factor X1
Rh-123	Rhodamine 123
ROS	Reactive oxygen species
RT-PCR	Real-time quantitative polymerase chain reaction
RXR	Retinoid X receptor
SKOV-3	Human ovarian adenocarcinoma
SKOV-3/Dox	Human ovarian adenocarcinoma, doxorubicin-resistant subline
SOX9	SRY-box transcription factor 9
SOX10	SRY-box transcription factor 10
SRC-1	Steroid receptor coactivator-1
SW620	Human colon cancer
VER	Verapamil
WIPO	World Intellectual Property Organization
WNT/β-catenin	Wingless-related integration site/β-catenin
WT1	Wilms’ tumor suppressor gene
W1PR	Human ovarian cancer, paclitaxel-resistant subline (primary cell line)
W1TR	Human ovarian cancer, topotecan-resistant subline (primary cell line)
