# Supplementary material for: Targeting Multidrug Resistance in Cancer: Impact of Retinoids, Rexinoids, and Carotenoids on ABC Transporters
Source: Int J Mol Sci. 2025 Nov 18;26(22):11157. doi: 10.3390/ijms262211157 (PMC12653979; doi:10.3390/ijms262211157)

Targeting Multidrug Resistance in Cancer: Impact of Retinoids, Rexinoids, and Carotenoids on ABC Transporters

Martina Čižmáriková [1,*,†], Viktória Háziková [1,†], Radka Michalková [1,*], Ondrej Franko [1], Beáta Lešková [1], Atila David Homolya [2], Juliana Gabzdilová [3] and Peter Takáč, Jr. [4]

[1] Department of Pharmacology, Faculty of Medicine, Pavol Jozef Šafárik University, 040 11 Košice, Slovakia; viktoria.hazikova@student.upjs.sk (V.H.); ondrej.franko@student.upjs.sk (O.F.); beata.leskova@student.upjs.sk (B.L.)
[2] Department of Radiotherapy and Oncology, East Slovakia Institute of Oncology, 041 91 Košice, Slovakia; atila.david.homolya@student.upjs.sk
[3] Department of Hematology and Oncohematology, Louis Pasteur University Hospital, Faculty of Medicine, Pavol Jozef Šafárik University, 040 11 Košice, Slovakia; juliana.gabzdilova@upjs.sk
[4] Department of Pharmacology and Toxicology, University of Veterinary Medicine and Pharmacy, Komenského 73, 041 81 Košice, Slovakia; peter.takac@uvlf.sk
* Correspondence: martina.cizmarikova@upjs.sk (M.Č.); radka.michalkova@upjs.sk (R.M.)
† These authors contributed equally to this work.

Figure S1. Chemical structures of retinoids. *Note 1:* For compound IIF, patent WIPO W000/117143, structural information was not available in the accessible resources and databases, therefore the corresponding structure is not provided.

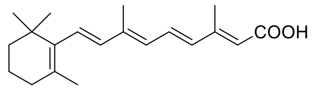

All-*trans*-retinoic acid

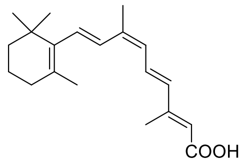

9-*Cis*-retinoic acid

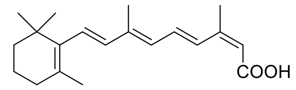

13-*Cis*-retinoic acid

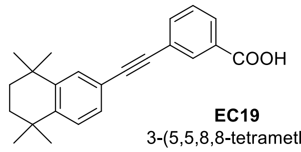

EC19
3-(5,5,8,8-tetramethyl-5,6,7,8-tetrahydro-naphthalen-2-ylethynyl)benzoic acid

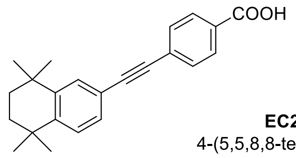

EC23
4-(5,5,8,8-tetramethyl-5,6,7,8-tetrahydro-naphthalen-2-ylethynyl)benzoic acid

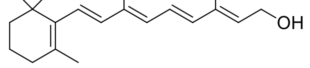

Retinol
Vitamin A

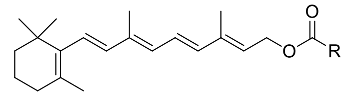

Retinyl acetate, R=CH_3
Retinyl palmitate, R=$C_{15}H_{31}$
Retinyl propionate, R=CH_3CH_2

Figure S2. Chemical structure of rexinoid.

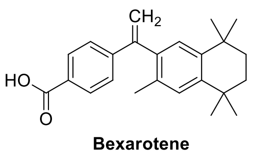

Bexarotene

Figure S3. Chemical structures of carotenoids. *Note 1:* Carotenes are depicted in blue, xantophylls in black and apocarotenoids in green. *Note 2 (for crocetin):* x = number of glycolic acid units, y = number of lactic acid units. *Note 3:* For fetoxanthin and violeoxanthin, structural information was not available in the accessible resources and databases, therefore the corresponding structures are not provided.

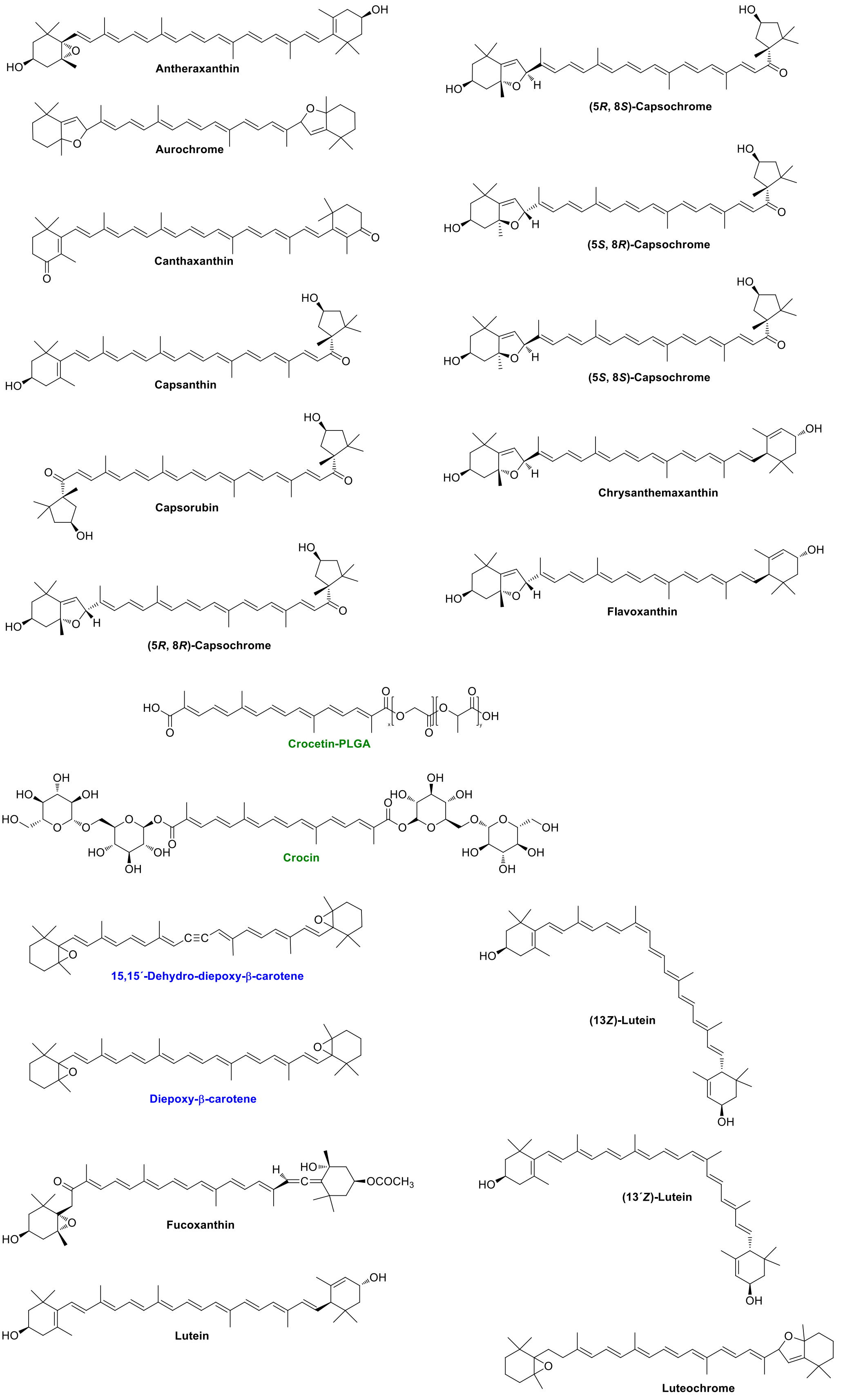

Figure S3 continued.

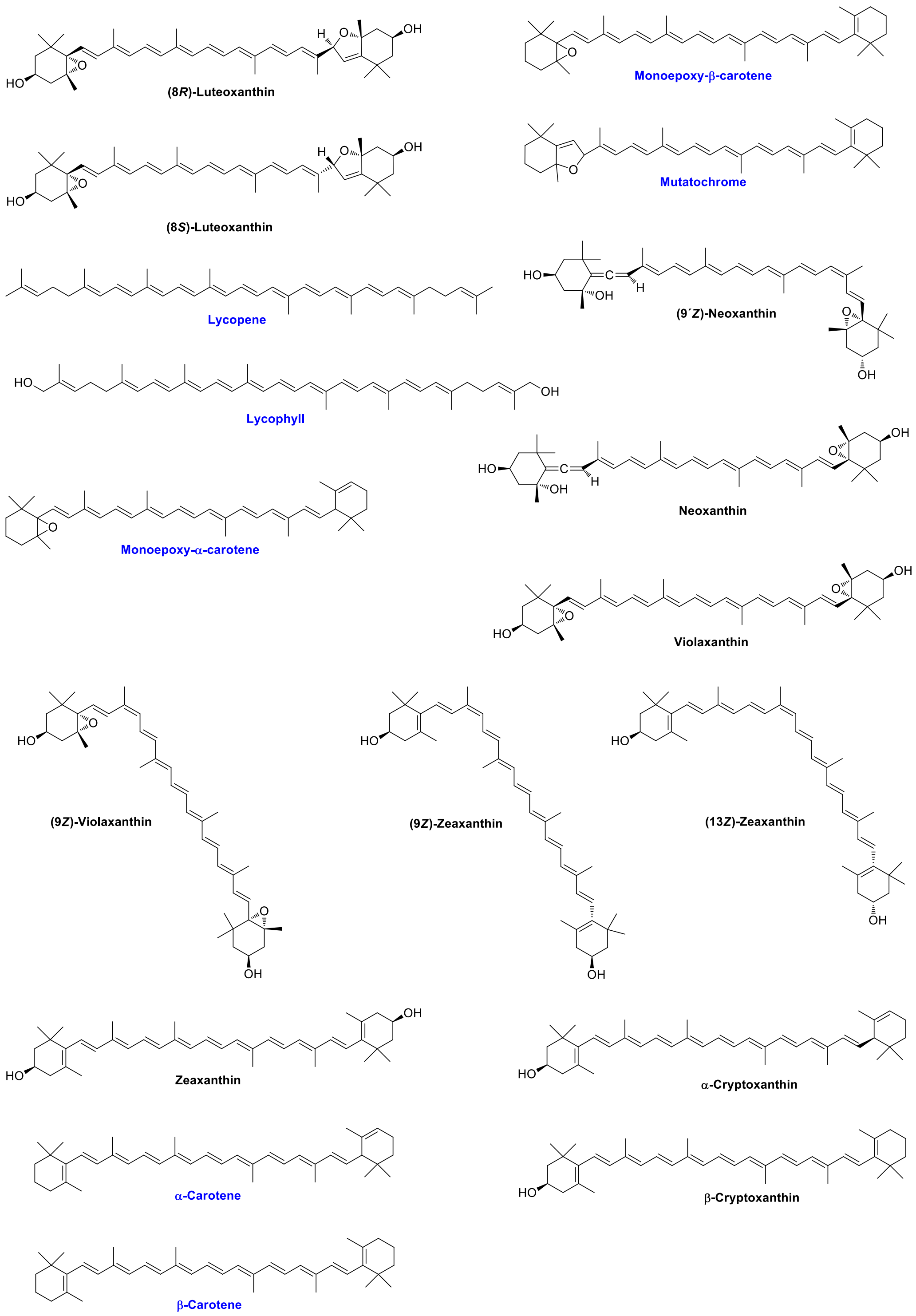

Supplement: Supplementary file 1 [file ijms-26-11157-s001.zip › ijms-3955988-supplementary.pdf]
